# Synthesis, Structural Confirmation, and Biosynthesis of 22-OH-PD1_n-3 DPA_ †

**DOI:** 10.3390/molecules24183228

**Published:** 2019-09-05

**Authors:** Jannicke Irina Nesman, Karoline Gangestad Primdahl, Jørn Eivind Tungen, Fransesco Palmas, Jesmond Dalli, Trond Vidar Hansen

**Affiliations:** 1 Department of Pharmacy, Section of Pharmaceutical Chemistry, University of Oslo, P.O. Box 1068 Blindern, N-0316 Oslo, Norway; 2 Lipid Mediator Unit, William Harvey Research Institute, Barts and The London School of Medicine, Queen Mary University of London, Charterhouse Square, London EC1M 6BQ, UK; 3 Centre for Inflammation and Therapeutic Innovation, Queen Mary University of London, London E1 4NS, UK

**Keywords:** natural products, stereoselective synthesis, structural elucidation, biosynthesis, protectins, 22-OH-PD1_n-3 DPA_, specialized pro-resolving mediators, polyunsaturated fatty acids, omega oxidation

## Abstract

PD1_n-3 DPA_ belongs to the protectin family of specialized pro-resolving lipid mediators. The protectins are endogenously formed mediators that display potent anti-inflammatory properties and pro-resolving bioactivities and have attracted interest in drug discovery. However, few studies have been reported of the secondary metabolism of the protectins. To investigate the metabolic formation of the putative C22 mono-hydroxylated product, coined 22-OH-PD1_n-3 DPA_, a stereoselective synthesis was performed. LC/MS-MS data of synthetic 22-OH-PD1_n-3 DPA_ matched the data for the biosynthetic formed product. Cellular studies revealed that 22-OH-PD1_n-3 DPA_ is formed from n-3 docosapentaenoic acid in human serum, and we confirmed that 22-OH-PD1_n-3 DPA_ is a secondary metabolite produced by ω-oxidation of PD1_n-3 DPA_ in human neutrophils and in human monocytes. The results reported are of interest for enabling future structure–activity relationship studies and provide useful molecular insight of the metabolism of the protectin class of specialized pro-resolving mediators.

## 1. Introduction

**Figure 1:**
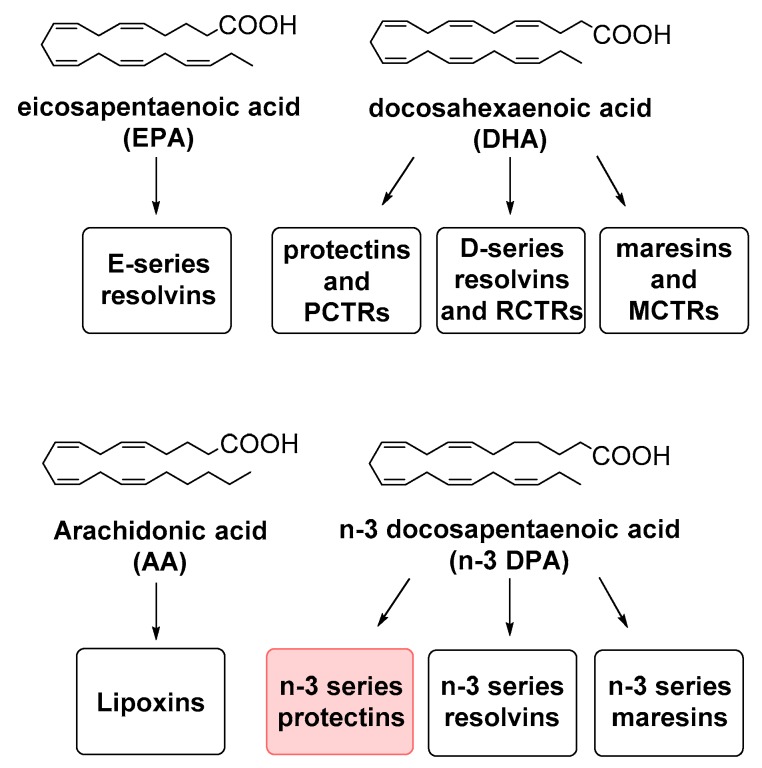
Overview of families of specialized pro-resolving mediators (SPMs) derived from eicosapentaenoic acid (EPA), docosahexaenoic acid (DHA), arachidonic acid (AA), and n-3 docosapentaenoic acid (DPA).

Inflammation is divided into acute inflammation, which by nature is self-resolving, and chronic inflammation, which occurs over an extended time period and does not resolve [1]. Uncontrolled, excessive acute and chronic unresolved inflammation may develop into several diseases, such as cardiovascular disease, cancer, rheumatoid arthritis, and neurological disorders, e.g., Parkinson‘s disease and Alzheimer’s disease [2]. Over the last century, inflammation has been the topic of numerous studies at the biomolecular and the cellular levels [3]. These efforts have resulted in the identification of several chemical mediators, such as peptides, oxygenated polyunsaturated fatty acids (PUFAs), chemokines, and cytokines that initiate, modulate, and reduce acute inflammatory processes [4]. Today, various drugs—mostly inhibitors—that reduce the effects of inflammatory processes are available [2,5]. Resolution of inflammation was earlier believed to be a passive process [1,2]. However, recent studies have established that the resolution phase of inflammation and the return to physiology (homeostasis) are regulated by active and enzymatic formation of several novel families of oxygenated PUFAs [4,6]. These endogenously formed compounds have been coined specialized pro-resolving mediators (SPMs) and are biosynthesized in the presence of cyclooxygenase and lipoxygenase enzymes from the dietary n-3 PUFAs eicosapentaenoic acid (EPA) and docosahexaenoic acid (DHA) (Figure 1) [6]. The E-series resolvins origin from EPA, the D-series resolvins, protectins and maresins, as well as the recently described sulfido-conjugates of resolvins (RCTRs), protectins (PCTRs), and maresins (MCTRs), are all biosynthesized from DHA [4]. Lipoxins are biosynthetically formed from arachidonic acid (AA) [3,5]. SPMs and their bioactions are considered to constitute a biomedical paradigm shift [5,7] with numerous interesting bioactivities reported [8].

SPMs display highly potent agonist effects in vivo, often in the low nanomolar range, by acting as ligands on individual G-protein coupled receptors (GPCRs) [9]. Approximately 35% of all approved non-biological drugs target GPCRs [10]. Moreover, SPMs are excellent biomolecular templates for the development of new, small molecular anti-inflammatory drugs and immunoresolvents [5,9], and some SPMs have entered initial clinical trial programs [9].

**Figure 2:**
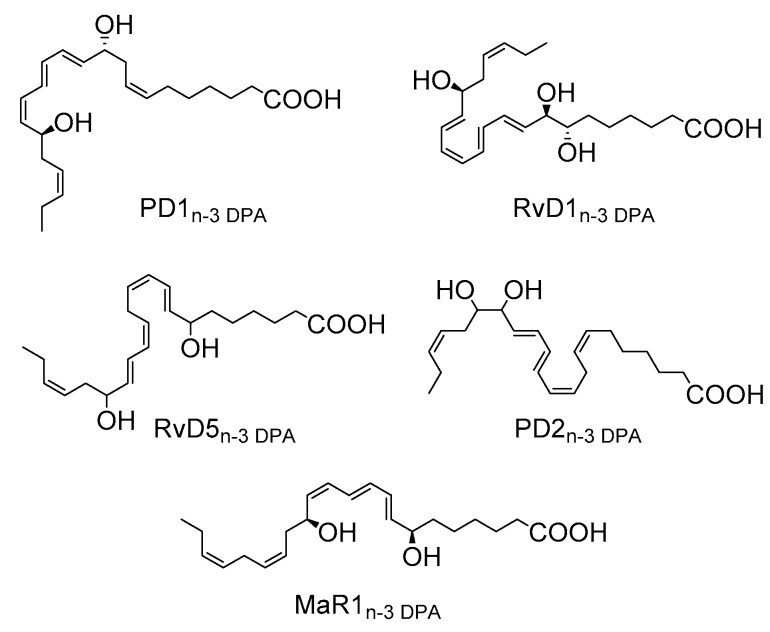
Chemical structures of some n-3 DPA derived SPMs. Where known, the absolute configuration is included.

New SPMs biosynthesized from the PUFA n-3 docosapentaenoic acid (n-3 DPA, **1**) were recently reported (Figure 2) [11,12]. N-3 DPA is formed from EPA and is also an intermediate in the biosynthesis of DHA [13]. Among these novel SPMs, PD1_n-3 DPA_ (**2**) has been the topic of detailed biological investigations [11,14,15,16,17,18,19].

**Scheme 1:**
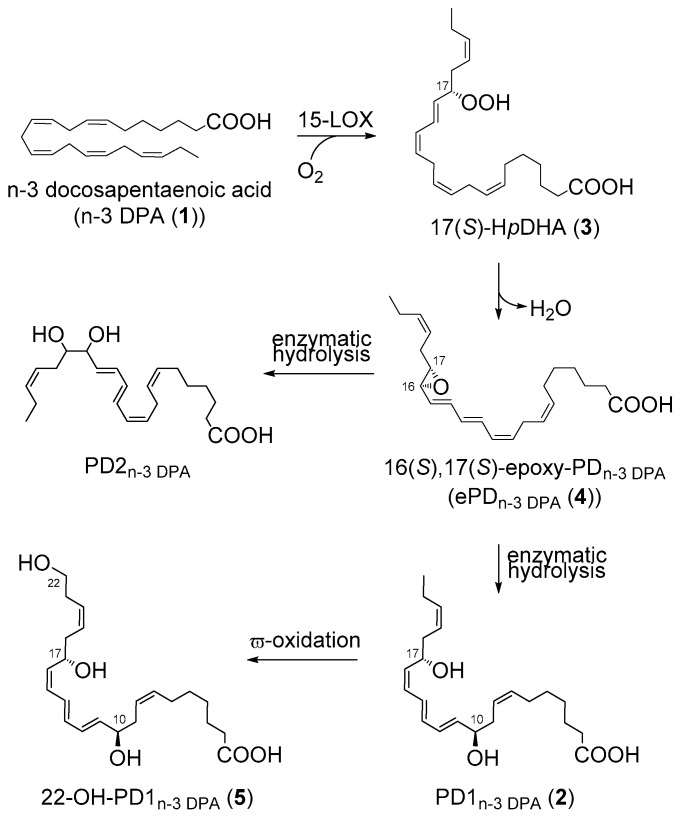
Established biosynthesis of **2** [20,21] and putative formation of 22-OH-PD1_n-3 DPA_ (**5**).

The biosynthesis of **2 [11]** was recently established [20,21], as presented in Scheme 1. First, a 17-lipoxygenation of n-3 DPA produces 17(*S*)-hydroperoxy-7*Z*,10*Z*,13*Z*,15*E*,19*Z*-docosapentaenoic acid [17(*S*)-H*p*DPA, **3**] that is converted into the epoxide intermediate 16(*S*),17(*S*)-epoxy PD_n-3 DPA_ (**4**), named ePD_n-3 DPA_. Epoxide **4** is then hydrolyzed to PD1_n-3 DPA_ (**2**) by an unknown hydrolase (Scheme 1). Further metabolism of **2** should result in the formation of 22-OH-PD1_n-3 DPA_ (**5**).

PUFAs and their oxygenated products undergo oxidative metabolism by eicosaoxidoreductase and cytochrome P450 (CYP) enzymes [6,22,23]. The ω-oxidative metabolism has been reported for protectin D1 (PD1) [24,25,26]. Of interest, in contrast to the ω-oxidation product of leukotriene B_4_ (LTB_4_), 20-OH-LTB_4_ [22,23], the PD1 metabolite 22-OH-PD1 [24] exhibited potent pro-resolving and anti-inflammatory bioactions in the nanomolar range [25]. Hence, we became interested in synthesizing **5** in a stereoselective manner and utilizing the synthetic material in biosynthetic investigations of the conversion of **1** and **2** into **5**.

## 2. Results and Discussion

### 2.1. Synthesis of 22-OH-PD1_n-3 DPA_

**Scheme 2:**
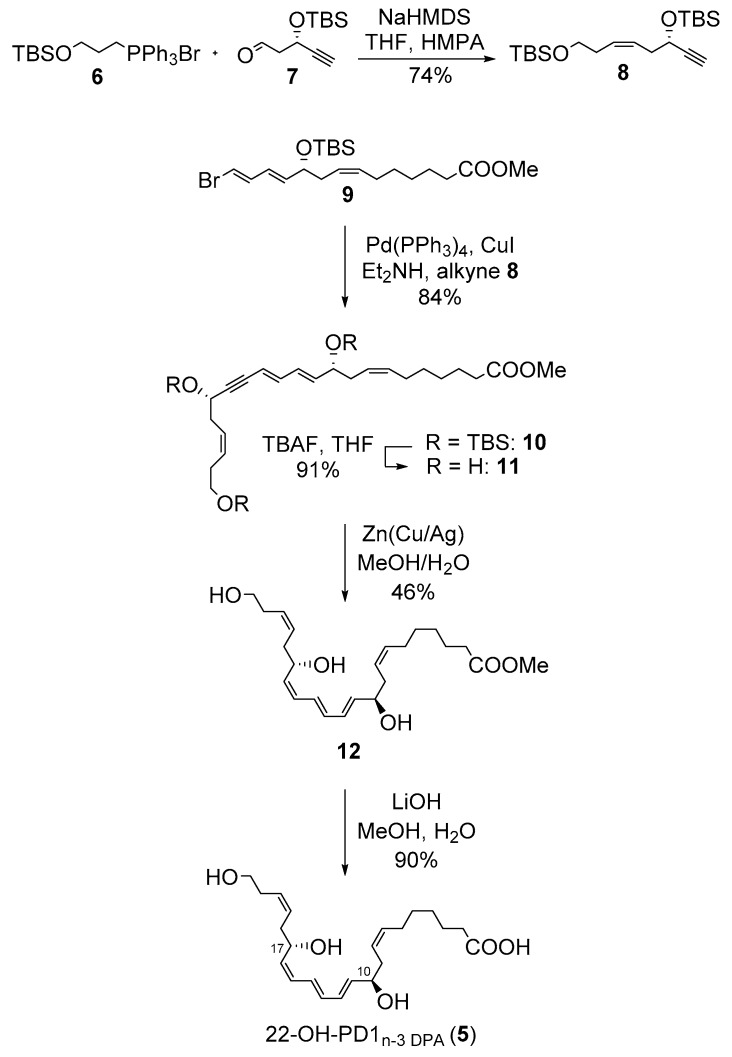
Synthesis of 22-OH-PD1_n-3 DPA_ (**5**).

To obtain stereochemically pure **5**, we first synthesized the Wittig-salt **6** from commercially available (3-bromopropoxy)-*tert*-butyldimethylsilane. The ylide of **6** was produced in the presence of sodium *bis*(trimethylsilyl)amide (NaHMDS) in THF/hexamethylphosphoric acid triamide (HMPA) and then reacted with the known aldehyde **7** [27] in a highly *Z-*selective Wittig reaction [28,29,30,31,32] (Scheme 2). This afforded *bis*-protected diol **8** in 74% isolated yield. The known vinylic bromide **9** was synthesized as earlier reported [14]. The two fragments **8** and **9** were then reacted in a Sonogashira reaction [(Pd(PPh_3_)_4_ (3 mol%) in Et_2_NH and in the presence of CuI (5 mol%)]. After column chromatography, isomeric pure **10** was obtained in 84% yield. Global deprotection using tetrabutylammonium fluoride (TBAF) (7.5 eq.) in THF at −78 °C afforded the triol **11** in 91% isolated yield. The *Z*-selective reduction of the internal alkyne in **11** proved challenging compared to similar systems [14,33,34]. Gratifyingly, the Boland reduction method [35] afforded the methyl ester **12** in 46% isolated yield after careful and repeated purifications by column chromatography. The chemical purity and the stereochemical integrity of **12** were validated using NMR and HPLC analyses, reaching > 95% purity (Supporting Information). Finally, mild saponification of the methyl ester in **12** afforded the target compound **5** in 90% isolated yield and in > 94% chemical purity based on NMR and HPLC analyses (Supporting Information). The geometrical configuration of the *E,E,Z*-triene moiety in **5** was assigned based on ^1^H NMR and UV experiments as well as by analogy with literature [14]. The coupling constants from the ^1^H NMR data were determined as 14.3 Hz, 13.9 Hz, and 11.4 Hz. The UV spectra of synthetic material of **5** showed absorbance peaks (λ_max_^EtOH^) at 262, 271, and 282 nm, which is in agreement with the UV absorption profile of a conjugated triene double bond system [11].

### 2.2. Matching of Synthetic 22-OH-PD1_n-3 DPA_ with Material Formed in Human Serum

**Figure 3:**
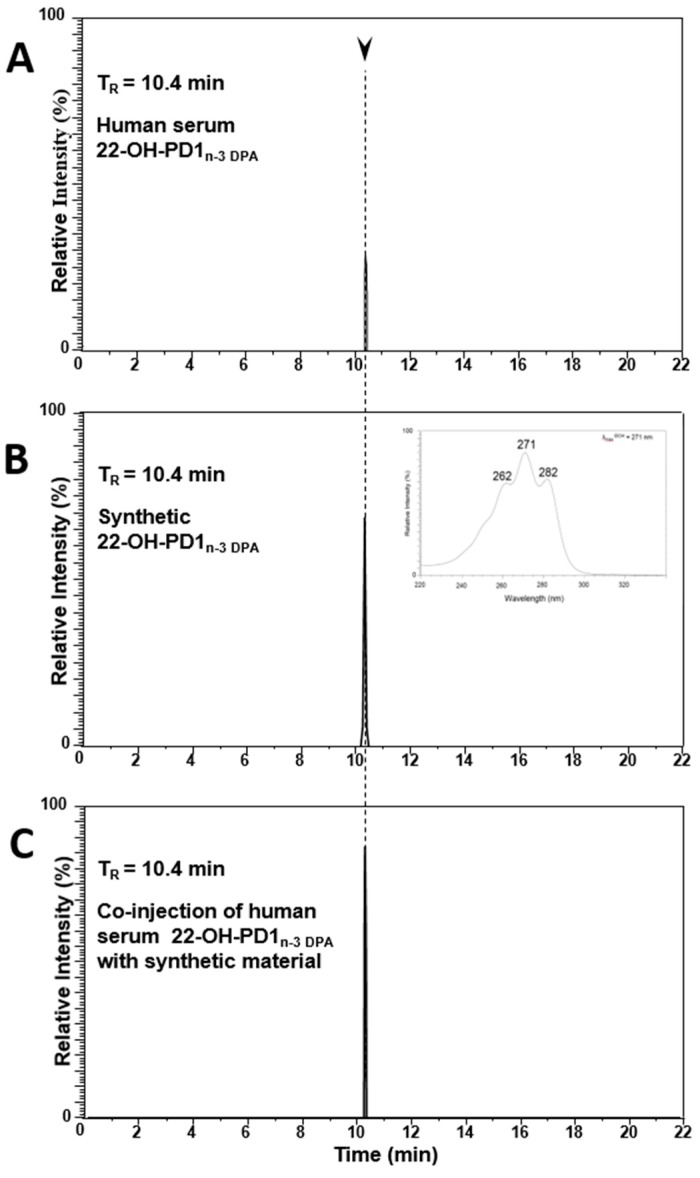
Multiple reaction monitoring (MRM) chromatogram of the products from (**A**) endogenous 22-OH-PD1_n-3 DPA_ (**5**) produced in human serum; (**B**) synthetic material of **5**; (**C**) co-injection of endogenous and synthetic material.

In order to obtain evidence that our synthetic material matched the authentic product of 22-OH-PD1_n-3 DPA_ (**5**), we assessed whether the physical properties of synthetic (**5**) matched with those of the product found in human serum [36]. Using liquid chromatography tandem mass spectrometry (LC/MS-MS) [37], we found that endogenous 22-OH-PD1_n-3 DPA_ (**5**) gave a retention time (T_R_-value) of 10.4 min using multiple reaction monitoring (MRM) (Figure 3A). Of note, synthetic **5** displayed the similar chromatographic behavior as the endogenous material (Figure 3B), and when these products were co-injected, they gave one peak in the MRM chromatogram with T_R_ = 10.4 min (Figure 3C).

The MS-MS spectra obtained from both endogenous **5** and synthetic **5** displayed essentially identical MS-MS fragmentation spectra with the following fragments assigned: *m*/*z* 377 = M − H, *m*/*z* 359 = M − H − H_2_O, *m*/*z* 341 = M − H − 2H_2_O, *m*/*z* 323 = M − H − 3H_2_O, *m*/*z* 297 = M − H − 2H_2_O − CO_2_, *m*/*z* 245 = 263 − H_2_O, *m*/*z* 165 = 183 − H_2_O and *m*/*z* 139 = 183 − CO_2_ (Supporting Information).

Overall, these results confirmed the structure of **5,** as depicted in Scheme 2, to be (7*Z*,10*R*,11*E*,13*E*,15*Z*,17*S*,19*Z*)-10,17,22-trihydroxydocosa-7,11,13,15,19-pentaenoic acid.

### 2.3. PD1_n-3 DPA_ is A Precursor in the Biosynthesis of 22-OH-PD1_n-3 DPA_ in Human Neutrophils

**Figure 4:**
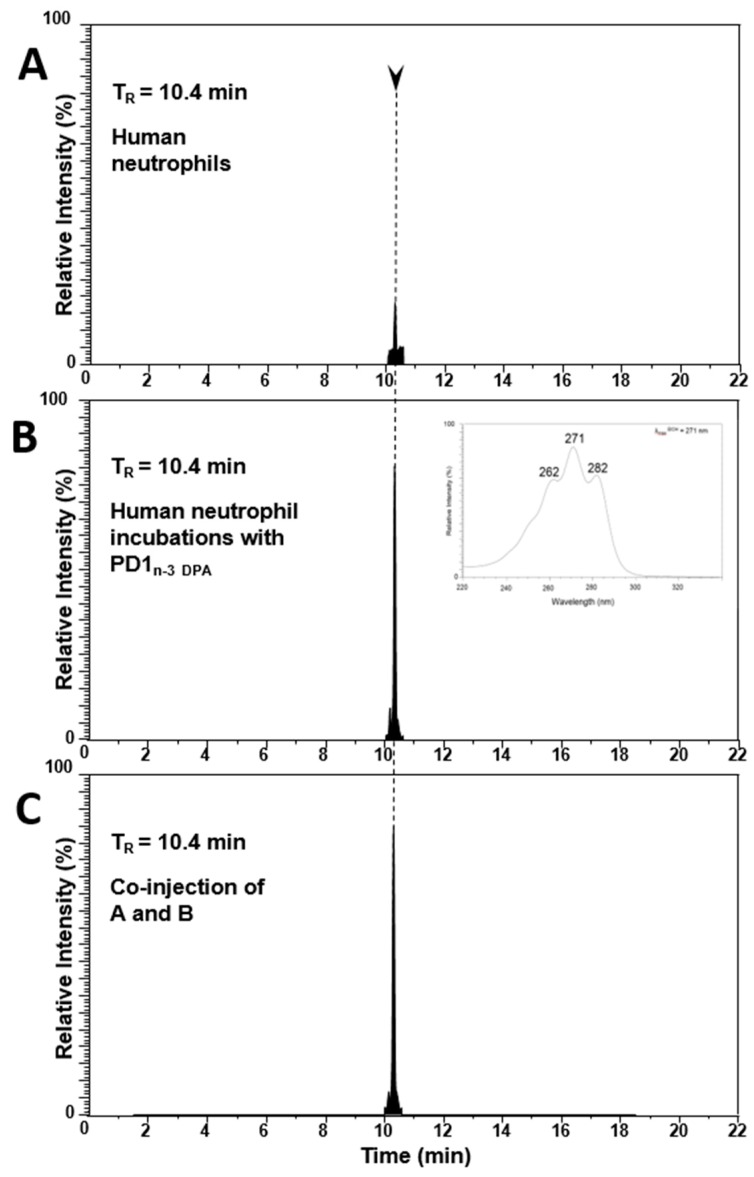
MRM chromatogram of the products from (**A**) human neutrophils incubated with vehicle; (**B**) human neutrophils incubated with PD1_n-3 DPA_ (**2**); (**C**) co-injection of samples obtained from (**A**,**B**).

First, we investigated if human neutrophils produced 22-OH-PD1_n-3 DPA_ (**5**) (Figure 4A). Human neutrophils were isolated from whole blood and profiled by means of LC/MS-MS. The MRM-chromatogram displayed a peak with T_R_ = 10.4 min (Figure 4A), and an MS/MS spectrum for the product under this peak matched that of the material identified in human serum and synthetic **5** (Supporting Information). Next, we determined whether PD1_n-3 DPA_ (**2**) was a precursor of 22-OH-PD1_n-3 DPA_ (**5**). The latter was obtained from earlier synthetic work [14]. For this purpose, we incubated **2** (10 nM) with human neutrophils, and the resulting product(s) profile was assessed using LC-MS/MS, searching targeting ion pairs with *m*/*z* 377 > 261 [36] in the MRM chromatogram. Here, we found that the intensity of the peak at T_R_ = 10.4 min corresponding to 22-OH-PD1_n-3 DPA_ was markedly increased when compared to the intensity of the peak obtained with human neutrophils alone (Figure 4B). Furthermore, the co-injection of samples from both experiments gave one single sharp peak at T_R_ = 10.4 min (Figure 4C). Overall, these experiments proved that PD1_n-3 DPA_ (**2**) was converted to 22-OH-PD1_n-3 DPA_ (**5**) by human neutrophils.

### 2.4. Biosynthesis of 22-OH-PD1_n-3 DPA_ from PD1_n-3 DPA_ in Human Monocytes

**Figure 5:**
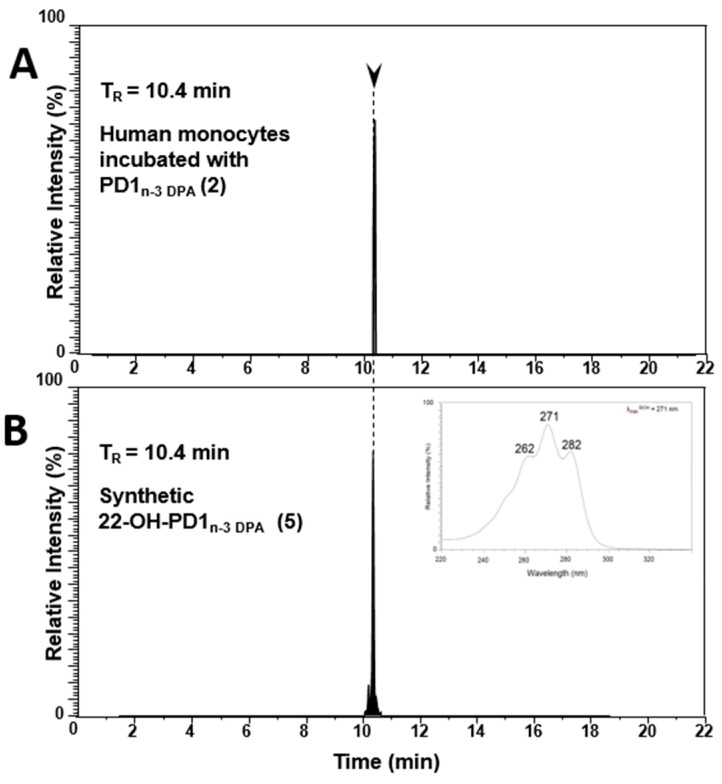
PD1_n-3 DPA_ (**2**) was converted to 22-OH-PD1_n-3 DPA_ (**5**) by human monocytes. Multiple reaction monitoring chromatograms for *m*/*z* 377 > 361 of the products obtained from (**A**) human monocytes incubated with PD1_n-3 DPA_; (**B**) synthetic 22-OH-PD1_n-3 DPA_.

In order to gain additional confidence for the direct biosynthetic formation of 22-OH-PD1_n-3 DPA_ (**5**) from PD1_n-3 DPA_ (**2**), SPM **2** was incubated (10 nM) with human monocytes. Again, data from LC/MS-MS experiments showed that 22-OH-PD1_n-3 DPA_ (**5**) was indeed formed from PD1_n-3 DPA_ (**2**) by human monocytes, since identical retention times were observed (Figure 5). In addition, the MS/MS-data of **5** from this experiment were in agreement with the data obtained from synthetic material of **5** (Supporting Information).

## 3. Materials and Methods

General: Unless otherwise stated, all commercially available reagents and solvents were used in the form they were supplied without any further purification. The stated yields were based on isolated material. All reactions were performed under an argon atmosphere using Schlenk techniques. Reaction flasks were covered with aluminum foil during reactions and storage to minimize exposure to light. Thin layer chromatography was performed on silica gel 60 F_254_ aluminum-backed plates fabricated by Merck, Darmstadt, Germany. Flash column chromatography was performed on silica gel 60 (40–63 μm) produced by Merck. NMR spectra were recorded on a Bruker AVI600 by Bruker, Billerica, MA, USA, a Bruker AVII400, or a Bruker DPX300 spectrometer at 600 MHz, 400 MHz, or 300 MHz, respectively, for ^1^H NMR and at 150 MHz, 100 MHz, or 75 MHz, respectively, for ^13^C NMR. Coupling constants (*J*) are reported in hertz, and chemical shifts are reported in parts per million (δ) relative to the central residual protium solvent resonance in ^1^H NMR (CDCl_3_ = δ 7.26, DMSO-*d*_6_ = δ 2.50, and MeOD = δ 3.31) and the central carbon solvent resonance in ^13^C NMR (CDCl_3_ = δ 77.00 ppm, DMSO-*d*_6_ = δ 39.43, and MeOD = δ 49.00). Optical rotations were measured using a 1 mL cell with a 1.0 dm path length on a Perkin Elmer 341 polarimeter by Perkin Elmer, Waltham, MA, USA. Mass spectra were recorded at 70 eV on Micromass Prospec Q or Micromass QTOF 2 W spectrometer by Miltham, MA, USA, using electrospray ionization (ESI) as the method of ionization. High-resolution mass spectra were recorded at 70 eV on Micromass Prospec Q or Micromass QTOF 2W spectrometer using ESI as the method of ionization. HPLC analyses were performed using a C18 stationary phase (Eclipse XDB-C18, 4.6 × 250 mm, particle size 5 μm, from Agilent Technologies, Santa Clara, CA, USA), applying the conditions stated. The UV/VIS spectra were recorded using an Agilent Technologies Cary 8485 UV-VIS spectrophotometer using quartz cuvettes.

### 3.1. Synthesis of Compounds

#### 3.1.1. Bromo(3-((*tert*-butyldimethylsilyl)oxy)propyl)triphenyl-^λ^l5-phosphane (**6**)

Commercially available (3-bromopropoxy)-*tert*-butyldimethylsilane (1.09 g, 4.32 mmol, 1.00 eq.) and PPh_3_ (1.25 g, 4.75 mmol, 1.10 eq.) in toluene (5.0 mL) were heated to reflux for 16 h. The reaction mixture was cooled to rt and extracted with Et_2_O (3 × 25 mL). The solvent was removed in vacuo to give a white, cloudy liquid. This liquid was purified by column chromatography on silica using pure dichloromethane as eluent until all excess PPh_3_ was eluted and there was 7% MeOH in CH_2_Cl_2_. The purified product was concentrated in vacuo to afford the title compound as a white solid in 76% yield (1.70 g). All spectroscopic and physical data were in agreement with those reported in the literature [38]. ^1^H NMR (400 MHz, CDCl_3_) δ 7.89–7.75 (m, 9H), 7.74–7.64 (m, 6H), 3.96–3.80 (m, 4H), 1.97–1.83 (m, 2H), 0.85 (s, 9H), 0.03 (s, 6H); ^13^C NMR (101 MHz, CDCl_3_) δ 135.1 (d, ^4′^*J*_CP_ = 3.0 Hz), 133.9 (d, ^3′^*J*_CP_ = 9.9 Hz), 130.6 (d, ^2′^*J*_CP_ = 12.5 Hz), 118.6 (d, ^1′^*J*_CP_ = 86.1 Hz), 61.9 (d, ^3^*J*_CP_ = 16.9 Hz), 26.2 (d, ^2^*J*_CP_ = 4.1 Hz), 26.1 (3C), 19.1 (d, ^1^*J*_CP_ = 52.6 Hz), 18.3, 5.2 (2C); thin layer chromatography (TLC) (MeOH/CH_2_Cl_2_ 1:20, KMnO_4_ stain) R*_f_* = 0.18; Mp: 137–139 °C.

#### 3.1.2. (*S*,*Z*)-5-Ethynyl-2,2,3,3,12,12,13,13-octamethyl-4,11-dioxa-3,12-disilatetradec-7-ene (**8**)

Wittig salt **6** (298 mg, 0.578 mmol, 1.00 eq.) was dissolved in THF (8.0 mL) and HMPA (0.45 mL). The solution was cooled to −78 °C followed by dropwise addition of NaHMDS (0.6 M in toluene, 0.95 mL, 0.986 eq.). The reaction mixture was stirred for 50 min, upon which it was warmed to rt and re-cooled to −78 °C. Aldehyde **7** (145 mg, 0.683 mmol, 1.20 eq.) in THF (1.0 mL) was added dropwise to the reaction mixture. After two hours, the reaction mixture was slowly allowed to warm to rt and then quenched with phosphate buffer (4.7 mL, pH = 7.2). The phases were separated, and the aqueous layer was extracted with Et_2_O (2 × 4.0 mL). The combined organic layers were dried (Na_2_SO_4_), and the solvent was removed by rotary evaporation. The crude product was filtered through a silica plug (hexane/EtOAc, 9:1) and concentrated in vacuo to afford compound **8** as a clear oil in 74% yield (157 mg); [α]${}_{D}^{20}$ = −15.0 (c = 1.33, CHCl_3_); ^1^H NMR (400 MHz, CDCl_3_) δ 5.59–5.49 (m, 2H), 4.34 (td, *J* = 6.5, 2.1 Hz, 1H), 3.61 (t, *J* = 7.0 Hz, 2H), 2.45 (t, *J* = 6.0 Hz, 2H), 2.38 (d, *J* = 2.1 Hz, 1H), 2.30 (q, *J* = 6.7 Hz, 2H), 0.90 (d, *J* = 3.8 Hz, 18H), 0.13 (s, 3H), 0.11 (s, 3H), 0.05 (s, 6H); ^13^C NMR (101 MHz, CDCl_3_) δ 128.7, 126.3, 85.4, 72.3, 63.0, 62.8, 36.8, 31.5, 26.1, 25.9, 18.5, 18.4, −4.5, −4.9, −5.1; TLC (hexane/EtOAc 95:5, KMnO_4_ stain) R*_f_* = 0.75; HRMS: exact mass calculated for C_20_H_40_O_2_Si_2_Na [M + Na]^+^: 391.2459, found: 391.2459.

#### 3.1.3. Methyl (7*Z*,10*R*,11*E*,13*E*,17*S*,19*Z*)-10,17,22-tris((*tert*-butyldimethylsilyl)oxy) docosa-7,11,13,19-tetraen-15-ynoate (**10**)

To a solution of vinyl bromide **9** (182 mg, 0.409 mmol, 1.00 eq.) in Et_2_NH (0.8 mL) and benzene (0.3 mL) was added Pd(PPh_3_)_4_ (16.0 mg, 0.0140 mmol, 3.00 mol%), and the reaction was stirred for 45 min in the dark. CuI (4.00 mg, 0.0210 mmol, 5.00 mol%) dissolved in a minimal amount of Et_2_NH was added, followed by dropwise addition of alkyne **8** (160 mg, 0.434 mmol, 1.06 eq.) in Et_2_NH (0.8 mL). After 20 h of stirring at ambient temperature, the reaction was quenched with saturated aqueous NH_4_Cl (10 mL). Et_2_O (15 mL) was added, and the phases were separated. The aqueous phase was extracted with Et_2_O (2 × 15 mL), and the combined organic layers were dried (Na_2_SO_4_) before being concentrated in vacuo. The crude product was purified by column chromatography on silica (hexane/EtOAc, 95:5) to afford the title compound **10** as a clear oil in 84% yield (239 mg); [α]${}_{D}^{25}$ = −11.6 (c = 0.95, MeOH); ^1^H NMR (400 MHz, CDCl_3_) δ 6.50 (dd, *J* = 10.9, 15.5 Hz, 1H), 6.18 (dd, *J* = 10.9, 15.5 Hz, 1H), 5.76 (dd, *J* = 6.0, 15.1 Hz, 1H), 5.61–5.49 (m, 3H), 5.47–5.30 (m, 2H), 4.47 (td, *J* = 6.5, 1.8 Hz, 1H), 4.20–4.12 (m, 1H), 3.66 (s, 3H), 3.61 (t, *J* = 7.0 Hz, 2H), 2.49–2.41 (m, 2H), 2.32–2.16 (m, 6H), 2.01 (q, *J* = 7.1 Hz, 2H), 1.68–1.53 (m, 2H), 1.39–1.24 (m, 5H), 0.91–0.88 (m, 27H), 0.13 (s, 3H), 0.11 (s, 3H), 0.06–0.01 (m, 12H); ^13^C NMR (101 MHz, CDCl_3_) δ 174.4, 141.2, 139.3, 131.9, 128.6, 128.5, 126.6, 125.3, 110.6, 93.3, 83.2, 72.9, 63.6, 63.0, 51.6, 37.0, 36.4, 34.2, 31.5, 29.4, 29.0, 27.4, 26.1, 26.0, 26.0, 25.0, 18.5, 18.4, 18.4, −4.3, −4.3, −4.6, −4.8, −5.1; TLC (hexane/EtOAc 95:5, UV-VIS) R*_f_* = 0.33; HRMS: exact mass calculated for C_41_H_76_O_5_Si_3_Na [M + Na]^+^: 755.4891, found: 755.4893.

#### 3.1.4. Methyl (7*Z*,10*R*,11*E*,13*E*,17*S*,19*Z*)-10,17,22-trihydroxydocosa-7,11,13,19-tetraen-15-ynoate (**11**)

TBAF (1.0 M in THF, 1.07 mL, 1.07 mmol, 7.48 eq.) was added to a solution of TBS-protected alcohol **10** (105 mg, 0.143 mmol, 1.00 eq.) in THF (1.5 mL) at −78 °C. The reaction was stirred for 21 h before it was quenched with phosphate buffer (pH = 7.2, 3.5 mL). Brine (15 mL) and EtOAc (15 mL) were added, and the phases were separated. The aqueous phase was extracted with EtOAc (2 × 10 mL), and the combined organic layer was dried (Na_2_SO_4_) before being concentrated in vacuo. The crude product was purified by column chromatography on silica (hexane/EtOAc, 4:6) to afford the title compound as a clear oil. Yield: 51 mg (91%); [α]${}_{D}^{25}$ = −24.4 (c = 0.67, MeOH); ^1^H NMR (400 MHz, MeOD) δ 6.55 (dd, *J* = 15.5, 10.8 Hz, 1H), 6.27 (dd, *J* = 15.5, 10.8 Hz, 1H), 5.81 (dd, *J* = 15.3, 6.4 Hz, 1H), 5.69–5.63 (m, 1H), 5.60–5.53 (m, 2H), 5.51–5.36 (m, 2H), 4.44 (td, *J* = 6.6, 1.9 Hz, 1H), 4.12 (q, *J* = 7.0, 6.5 Hz, 1H), 3.65 (s, 3H), 3.56 (t, *J* = 6.8 Hz, 2H), 2.49–2.44 (m, 2H), 2.36–2.22 (m, 6H), 2.05 (q, *J* = 6.9 Hz, 2H), 1.61 (p, *J* = 7.4 Hz, 2H), 1.42–1.28 (m, 4H); ^13^C NMR (101 MHz, MeOD) δ 176.0, 142.5, 139.9, 132.9, 130.3, 129.7, 127.5, 126.1, 111.7, 93.8, 84.3, 72.8, 63.1, 62.6, 52.0, 37.0, 36.2, 34.8, 32.0, 30.3, 29.80, 28.2, 25.9; TLC (hexane/EtOAc 2:3, UV-VIS) R*_f_* = 0.22; HRMS: exact mass calculated for C_23_H_34_O_5_Na [M + Na]^+^: 413.2299, found: 413.2298.

#### 3.1.5. 22-OH-PD1_n-3 DPA_ Methyl Ester (**12**)

The Zn(Cu/Ag) mixture was prepared as described by Boland et al. [35]. Zinc dust (3.12 g) in degassed H_2_O (18.8 mL, pH = 7.0) was stirred under argon for 15 min before Cu(OAc)_2_ (312 mg) was added and stirred for an additional 15 min. AgNO_3_ (312 mg) was then added, and the reaction mixture was stirred for 30 min. The mixture was filtered and washed successively with H_2_O, MeOH, acetone, and Et_2_O before it was transferred to a flask containing alkyne **11** (50.0 mg, 0.128 mmol) in MeOH/H_2_O (1:3, 9.2 mL). The mixture was stirred at rt and monitored by TLC analysis. After 2 h, the reaction was judged complete, and the reaction mixture was filtered through a pad of Celite^®^ by Merck, Darmstadt, Germany, with Et_2_O. Water was added to the filtrate, and the layers were separated. The aqueous layer was extracted with Et_2_O (2 × 10 mL). The combined organic layers were washed with brine and dried (Na_2_SO_4_). The solvent was removed in vacuo, and the crude product was purified by column chromatography on silica gel (hexane/EtOAc/MeOH, 49:50:1) to afford the methyl ester **12** as a pale yellow oil in 46% yield (23 mg); [α]${}_{D}^{25}$ = −20.0 (c = 0.20, MeOH); ^1^H NMR (600 MHz, MeOD) δ 6.58–6.48 (m, 1H), 6.32–6.20 (m, 2H), 6.08 (t, *J* = 11.2 Hz, 1H), 5.75 (dd, *J* = 6.6, 14.5 Hz, 1H), 5.53–5.36 (m, 5H), 4.62–4.55 (m, 1H), 4.15–4.08 (m, 1H), 3.65 (s, 3H), 3.55 (t, *J* = 6.8 Hz, 2H), 2.43–2.36 (m, 1H), 2.35–2.18 (m, 7H), 2.09–1.99 (m, 2H), 1.61 (p, *J* = 7.4 Hz, 2H), 1.42–1.27 (m, 4H); ^13^C NMR (151 MHz, MeOD) δ 176.0, 138.1, 135.0, 134.8, 132.8, 131.4, 130.6, 129.1, 128.8, 128.1, 126.2, 73.1, 68.5, 62.6, 52.0, 36.6, 36.4, 34.8, 32.0, 30.3, 29.8, 28.2, 25.9; TLC (hexane/EtOAc/MeOH 49:50:1) R*_f_* = 0.22; HRMS: exact mass calculated for C_23_H_36_O_5_Na [M + Na]^+^: 415.2455, found: 415.2455; UV-VIS: λ_max_ (EtOH) = 269, 271, 282 nm. The purity (96%) was determined by HPLC analysis (Eclipse XDB-C18, MeOH/H_2_O 3:1, 1.0 mL/min); t_r_ = 32.6.

#### 3.1.6. 22-OH-PD1_n-3 DPA_ (**5**)

Solid LiOH (17.0 mg, 0.710 mmol, 31.0 eq.) was added to a solution of methyl ester **12** (9 mg, 0.0229 mmol, 1.00 eq.) dissolved in THF-MeOH-H_2_O (2:2:1, 2.8 mL) at 0 °C. The reaction mixture was stirred at 0 °C for three hours and then allowed to warm to rt. After 7.5 h, the solution was cooled to 0 °C, acidified with saturated aqueous NaH_2_PO_4_ (4.0 mL), and then EtOAc (4.0 mL) was added. The layers were separated, and the water phase was extracted with EtOAc (2 × 4.0 mL). The combined organic layers were dried (Na_2_SO_4_) before the solvent was removed in vacuo. Then, 22-OH-PD1_n-3 DPA_ (**5**) was obtained as a colorless oil after purification by column chromatography (MeOH/CH_2_Cl_2_, 1:9) R*_f_* = 0.22; yield: 10 mg (90%); [α]${}_{D}^{25}$ = −25.4 (c = 0.35, MeOH); ^1^H NMR δ 6.53 (dd, *J* = 13.9, 11.4 Hz, 1H), 6.32–6.21 (m, 2H), 6.08 (t, *J* = 11.4 Hz, 1H), 5.74 (dd, *J* = 14.3, 6.5 Hz, 1H), 5.56–5.44 (m, 3H), 5.43–5.36 (m, 2H), 4.63–4.55 (m, 1H), 4.12 (q, *J* = 6.6 Hz, 1H), 3.55 (t, *J* = 6.8 Hz, 2H), 2.44–2.36 (m, 1H), 2.36–2.20 (m, 7H), 2.06 (q, *J* = 7.0 Hz, 2H), 1.60 (p, *J* = 7.4 Hz, 2H), 1.41–1.32 (m, 4H); ^13^C NMR (151 MHz, MeOD) δ 178.0, 138.1, 135.0, 134.8, 132.9, 131.4, 130.6, 129.1, 128.8, 128.1, 126.2, 73.1, 68.5, 62.6, 36.6, 36.4, 35.3, 32.0, 30.4, 29.9, 28.3, 26.1; HRMS: exact mass calculated for C_22_H_34_O_5_Na [M + Na]^+^: 401.2298, found:401.2298; UV-VIS: λ_max_ (EtOH) = 269, 271, 282 nm. The purity (94%) was determined by HPLC analysis (Eclipse XDB-C18, MeOH/H_2_O/10mM acetic acid 65:20:20, 1.0 mL/min); T_r_ = 18.1.

### 3.2. Lipid Mediator Profiling

MeOH—two and four volumes, respectively—containing deuterium-labeled synthetic internal standards of *d*_4_-LTB_4_ (500 pg) and *d*_5_-RvE1 (100 pg) were added to cell incubations and human serum. Samples were stored at −40 °C until extraction. Prior to extraction, samples were centrifuged at 2500 rpm, 4 °C, for 10 min. Supernatants were then collected and concentrated to ~1.0 mL of MeOH content using a gentle stream of nitrogen gas (TurboVap LV system, Biotage, Uppsala, Sweden). Solid phase extraction was performed by means of the ExtraHera (Biotage) automated extraction system as follows. Aqueous HCl solution (pH = 3.5, 9.0 mL) was added to the samples, and the acidified solutions were loaded onto conditioned C18 500 mg 200-0050-B cartridges (Biotage). Prior to extraction, solid phase C18 cartridges were equilibrated with MeOH (3.0 mL) and H_2_O (6.0 mL). The extraction products were washed with H_2_O (4.0 mL) and hexane (5.0 mL) followed by product elution with 4.0 mL of methyl formate. Products were brought to dryness with a gentle stream of nitrogen (TurboVap LV, Biotage) and suspended in MeOH:H_2_O (50:50, *v*/*v*). Samples were centrifuged (2500 rpm, 4.0 °C, 5 min), and the supernatant was collected and centrifuged (9900 rpm, 4.0 °C, 10 sec). The collected supernatant was then subjected to LC/MS-MS, as described in reference [39].

### 3.3. Human Neutrophil Incubations

Human peripheral blood neutrophils were isolated from healthy volunteers using density-gradient Ficoll-Histopaque isolation. Volunteers gave written consent in accordance with a Queen Mary Research Ethics Committee (QMREC 2014:61) and the Helsinki declaration. Isolated neutrophils were suspended in PBS^+/+^ containing 10% bovine serum albumin (BSA) and incubated for 10 min (37 °C, pH = 7.45) prior to the addition of the indicated concentrations of n-3 DPA (**1**) and *Escherichia coli* (2.5 × 10^8^ cells/mL). Incubations were quenched using two volumes ice-cold methanol containing deuterium-labeled internal standards to facilitate quantification and identification. Products were extracted using C18 columns and quantified by LC/MS-MS metabololipidomics, as detailed above. The same experiment was repeated with synthetic **2**.

### 3.4. Human Serum Incubations

Human pool serum was purchased from Sigma-Aldrich (Poole, UK). Four volumes of methanol containing deuterium-labeled standards were added, and products were extracted using C18 column and profiled using LC/MS-MS metabololipidomics, as detailed above [39].

## 4. Conclusions

To summarize, 22-OH-PD1_n-3 DPA_ (**5**) was stereoselectively synthesized in six steps and with 38% yield from commercially available (3-bromopropoxy)-*tert*-butyldimethylsilane and known (*S*)-3-((*tert*-butyldimethylsilyl)oxy)pent-4-ynal (**7**). We herein established the exact configuration of the biomolecule **5**, and that **5** is an ω-oxidation metabolic product of the SPM PD1_n-3 DPA_ (**2**). In addition, we showed that 22-OH-PD1_n-3 DPA_ (**5**) is formed in human serum, human neutrophils, and by human monocytes. The results reported contribute to additional information and knowledge of the growing number of members of the SPM family of endogenously formed lipid mediators and their metabolism, which is of interest in regard to developing new immunoresolvents without immunosuppressive effects [5,37].

## Supplementary Materials

Electronic Supporting Information is available with ^1^H-, ^13^C-NMR, HPLC-chromatograms, UV-VIS, MS/MS and HRMS data of **5** and intermediates **6**–**11** and methyl ester **12**. See https://www.mdpi.com/1420-3049/24/18/3228/s1.

## References

[1] Rather L.J. (1971). Disturbance of function (function laesa): The legendary fifth cardinal sign of inflammation, added by Galen to the four cardinal signs of Celsus. Bull. N. Y. Acad. Med..

[2] Libby P. (2007). Inflammatory mechanisms: The molecular basis of inflammation and disease. Nutr. Rev..

[3] Levy B.D., Clish C.B., Schmidt B., Gronert K., Serhan C.N. (2001). Lipid mediator class switching during acute inflammation: Signals in resolution. Nat. Immunol..

[4] Tabas I., Glass C.K. (2013). Anti-inflammatory therapy in chronic disease: Challenges and opportunities. Science.

[5] Fullerton J.N., Gilroy D.W. (2016). Resolution of inflammation: A new therapeutic frontier. Nat. Rev. Drug Discov..

[6] Serhan C.N., Petasis N.A. (2011). Resolvins and protectins in inflammation resolution. Chem. Rev..

[7] Van Dyke T.E., Serhan C.N. (2003). Resolution of inflammation: A new paradigm for the pathogenesis of periodontal disease. J. Dent. Res..

[8] Serhan C.N. (2017). Treating inflammation and infection in the 21^st^ century: New hints from decoding resolution mediators and mechanisms. FASEB J..

[9] Serhan C.N., Chiang N. (2013). Resolution phase lipid mediators of inflammation: Agonists of resolution. Curr. Opin. Pharmacol..

[10] Lappano R., Maggiolini M. (2011). G protein-coupled receptors: Novel targets for drug discovery in cancer. Nat. Rev. Drug Discov..

[11] Dalli J., Colas R.A., Serhan C.N. (2013). Novel n-3 immunoresolvents: Structures and actions. Sci. Rep..

[12] Dalli J., Chiang N., Serhan C.N. (2015). Elucidation of novel 13-series resolvins that increase with atorvastatin and clear infections. Nat. Med..

[13] Kaur G., Cameron-Smith D., Garg M., Sinclair A.J. (2011). Docosapentaenoic acid (22:5n-3): A review of its biological effects. Prog. Lipid Res..

[14] Aursnes M., Tungen J.E., Vik A., Colas R., Cheng C.-Y.C., Dalli J., Serhan C.N., Hansen T.V. (2014). Total synthesis of the lipid mediator PD1_n-3 DPA_: Configural assignments and anti-inflammatory and pro-resolving actions. J. Nat. Prod..

[15] Hansen T.V., Vik A., Serhan C.N. (2019). The protectin family of specialized pro-resolving mediators: Potent immunoresolvents enabling innovative approaches to target obesity and diabetes. Front. Pharmacol..

[16] Hansen T.V., Dalli J., Serhan C.N. (2017). The novel lipid mediator PD1_n-3 DPA_: An overview of the structural elucidation, synthesis, biosynthesis and bioactions. Prostaglandins Other Lipid Mediat..

[17] Vik A., Dalli J., Hansen T.V. (2017). Recent advances in the chemistry and biology of anti-inflammatory and specialized pro-resolving mediators biosynthesized from n-3 docosapentaenoic acid. Bioorg. Med. Chem. Lett..

[18] Gobbetti T., Dalli J., Colas R.A., Canova D.F., Aursnes M., Bonnet D., Alric L., Vergnolle N., Deraison C., Hansen T.V. (2017). Protectin D1_n-3 DPA_ and resolvin D5_n-3 DPA_ are novel effectors of intestinal protection. Proc. Natl. Acad. Sci. USA.

[19] Frigerio F., Pasqualini G., Craparotta I., Marchini S., Van Vliet E.A., Foerch P., Vandenplas C., Leclercq K., Aronica E., Porcu L. (2018). n-3 Docosapentaenoic acid-derived protectin D1 promotes resolution of neuroinflammation and arrests epileptogenesis. Brain.

[20] Pistorius K., Souza P.R., De Matteis R., Austin-Williams S., Primdahl K.G., Vik A., Mazzacuva F., Colas R.A., Marques R.M., Hansen T.V. (2018). PD_n-3 DPA_ pathway regulates human monocyte differentiation and macrophage function. Cell Chem. Biol..

[21] Primdahl K.G., Tungen J.E., De Souza P.R.S., Colas R.A., Dalli J., Hansen T.V., Vik A. (2017). Stereocontrolled synthesis and investigation of the biosynthetic transformations of 16(*S*), 17(*S*)-epoxy-PD_n-3 DPA_. Org. Biomol. Chem..

[22] Sumimoto H., Minakami S. (1990). Oxidation of 20- hydroxyleukotriene B_4_ by human neutrophil microsomes. Role of aldehyde dehydrogenase and leukotriene B_4_ ω-hydroxylase (cytochrome P-450_LTB4_) in leukotriene B_4_ ω-oxidation. J. Biol. Chem..

[23] Divanovic S., Dalli J., Jorge-Nebert L.F., Flick L.M., Gálvez-Peralta M., Boespflug N.D., Stankiewicz T.E., Fitzgerald J.M., Somarathna M., Karp C.L. (2013). Contributions of the three CYP1 monooxygenases to pro-inflammatory and inflammation-resolution lipid mediator pathways. J. Immunol..

[24] Serhan C.N., Hong S., Gronert K., Colgan S.P., Devchand P.R., Mirick G., Moussignac R.-L. (2002). Resolvins. A family of bioactive products of omega-3 fatty acid transformation circuits initiated by aspirin treatment that counter proinflammation signals. J. Exp. Med..

[25] Tungen J.E., Aursnes M., Vik A., Colas R.A., Dalli J., Serhan C.N., Hansen T.V. (2014). Synthesis, anti-inflammatory and pro-resolving activities of 22-OH-PD1, a mono-hydroxylated metabolite of protectin D1. J. Nat. Prod..

[26] Tungen J.E., Aursnes M., Ramon S., Colas R.A., Serhan C.N., Olberg D.E., Nuruddin S., Willoch F., Hansen T.V. (2018). Synthesis of protectin D1 analogs: Novel pro-resolution and radiotracer agents. Org. Biomol. Chem..

[27] Tungen J.E., Aursnes M., Dalli J., Arnardottir H., Serhan C.N., Hansen T.V. (2014). Total synthesis of the anti-inflammatory and pro-resolving lipid mediator MaR_n-3 DPA_. Chem. Eur. J..

[28] Ramon S., Dalli J., Sanger J.M., Winkler J.W., Aursnes M., Tungen J.E., Hansen T.V., Serhan C.N. (2016). The protectin PCTR1 is produced by human M2 macrophages and enhances resolution of infectious inflammation. Am. J. Phatol..

[29] Primdahl K.G., Aursnes M., Tungen J.E., Hansen T.V., Vik A. (2015). An efficient synthesis of leukotriene B_4_. Org. Biomol. Chem..

[30] Tungen J.E., Aursnes M., Hansen T.V. (2015). Stereoselective synthesis of maresin 1. Tetrahedron Lett..

[31] Nolsøe J.M., Aursnes M., Tungen J.E., Hansen T.V. (2015). Dienals derived from pyridinium salts and their subsequent application in natural product synthesis. J. Org. Chem..

[32] Aursnes M., Tungen J.E., Vik A., Dalli J., Hansen T.V. (2014). Stereoselective synthesis of protectin D1: A potent anti-inflammatory and pro-resolving lipid mediator. Org. Biomol. Chem..

[33] Primdahl K.G., Aursnes M., Walker M.E., Colas R.A., Serhan C.N., Dalli J., Hansen T.V., Vik A. (2016). Synthesis of 13(*R*)-Hydroxy-7*Z*,10*Z*,13*R*,14*E*,16*Z*,19*Z* docosapentaenoic acid (13*R*-HDPA) and its biosynthetic conversion to the 13-series resolvins. J. Nat. Prod..

[34] Tungen J.E., Gerstmann L., Vik A., De Mateis R., Colas R.A., Dalli J., Chiang N., Serhan C.N., Kalesse M., Hansen T.V. (2019). Resolving inflammation: synthesis, configurational assignment, and biological evaluations of RvD1n− 3 DPA. Chem. Eur. J..

[35] Boland W., Schroer C.N., Sieler C.M., Feigel M. (1987). Stereospecific syntheses and spectroscopic properties of isomeric 2,4,6,8-undecatetraenes. New hydrocarbons from the marine brown alga *Giffordia mitchellae*. Part IV. Helv. Chim. Acta..

[36] Dalli J., Serhan C.N. (2012). Specific lipid mediator signatures of human phagocytes: Microparticles stimulate macrophage efferocytosis and pro-resolving mediators. Blood.

[37] Dalli J. (2017). Does promoting resolution instead of inhibiting inflammation represent the new paradigm in treating infections?. Mol. Aspects Med..

[38] Loiseau F., Simone J.-M., Carcache D., Bobal P., Neier R. (2007). Radical couplings as key steps for the preparation of derivatives of nonactic acid. Montash. Chem..

[39] Dalli J., Colas R.A., Walker M.E., Serhan C.N. (2018). Lipid mediator metabolomics via LC-MS/MS profiling and analysis. Methods Mol. Biol..

