# Supplementary material for: Synthesis, Structural Confirmation, and Biosynthesis of 22-OH-PD1n-3 DPA
Source: Molecules. 2019 Sep 5;24(18):3228. doi: 10.3390/molecules24183228 (PMC6767081; doi:10.3390/molecules24183228)
Supplement: Supplementary file 1 [file molecules-24-03228-s001.pdf]

Supporting Information for

Synthesis, Structural Confirmation and Biosynthesis of 22-OH-PD1$_{n\text{-}3\ DPA}$

Jannicke I. Nesman[†], Karoline G. Primdahl[†], Jørn E. Tungen[†], Francesco Palmas[‡], Jesmond Dalli[‡c] and Trond V. Hansen[†]*

[†]*School of Pharmacy, Department of Pharmaceutical Chemistry, University of Oslo, PO Box 1068 Blindern, N-0316 Oslo, Norway*

[‡]*Lipid Mediator Unit, William Harvey Research Institute, Barts and The London School of Medicine, Queen Mary University of London, Charterhouse Square, London EC1 M 6BQ, UK*

[*]*E-mail: t.v.hansen@farmasi.uio.no*

[c] *Centre for Inflammation and Therapeutic Innovation, Queen Mary University of London, London, UK*

Contents

General Information 1

Experimental Details 2

^{1}H NMR and ^{13}C NMR spectra of compounds 7

HPLC chromatograms 17

Lipid Mediator Metabololipidomics 18

Matching of synthetic 22-OH-PD1$_{n\text{-}3\ DPA}$ with material formed in human monocytes incubated with PD1$_{n\text{-}3\ DPA}$ 18

MS-MS fragmentation spectrums of 22-OH-PD1$_{n\text{-}3\ DPA}$ 19

UV-VIS spectra 20

References 20

General Information

Unless otherwise stated, all commercially available reagents and solvents were used in the form they were supplied without any further purification. The stated yields are based on isolated material. All reactions were performed under an argon atmosphere using Schlenk techniques. Reaction flasks were covered with aluminum foil during reactions and storage to minimize exposure to light. Thin layer

chromatography was performed on silica gel 60 F_{254} aluminum-backed plates fabricated by Merck. Flash column chromatography was performed on silica gel 60 (40-63 μm) produced by Merck. NMR spectra were recorded on a Bruker AVI600, Bruker AVII400 or a Bruker DPX300 spectrometer at 600 MHz, 400 MHz or 300 MHz respectively for ^{1}H NMR and at 150 MHz, 100 MHz or 75 MHz respectively for ^{13}C NMR. Coupling constants (*J*) are reported in hertz and chemical shifts are reported in parts per million (δ) relative to the central residual protium solvent resonance in ^{1}H NMR ($CDCl_3$ = δ 7.26, DMSO-d_6 = δ 2.50 and MeOD = δ 3.31) and the central carbon solvent resonance in ^{13}C NMR ($CDCl_3$ = δ 77.00 ppm, DMSO-d_6 = δ 39.43 and MeOD = δ 49.00). Optical rotations were measured using a 1 mL cell with a 1.0 dm path length on a Perkin Elmer 341 polarimeter. Mass spectra were recorded at 70 eV on Micromass Prospec Q or Micromass QTOF 2 W spectrometer using ESI as the method of ionization. High-resolution mass spectra were recorded at 70 eV on Micromass Prospec Q or Micromass QTOF 2W spectrometer using ESI as the method of ionization. HPLC-analyses were performed using a C18 stationary phase (Eclipse XDB-C18, 4.6 x 250 mm, particle size 5 μm, from Agilent Technologies), applying the conditions stated. The UV-VIS spectra were recorded using an Agilent Technologies Cary 8485 UV-VIS spectrophotometer using quartz cuvettes.

Experimental Details

(*S*)-4-isopropylthiazolidine-2-thione (14)

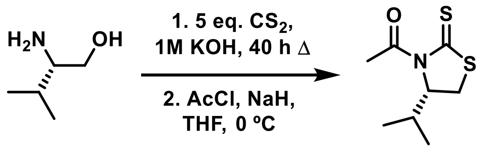

Nagao's chiral auxiliary **14**, was prepared from commercially available (*S*)-(+)-2-amino-3-methyl-1-butanol (**13**) as previously reported in the literature.[1-2] Yield: 67% over the two steps. All spectroscopic and physical data were in agreement with those reported in the literature.[2] $[\alpha]_D^{20}$ = 434 (c = 0.26, $CHCl_3$); **^{1}H NMR** (400 MHz, $CDCl_3$) δ 5.15 (ddd, *J* = 7.6, 6.2, 1.2 Hz, 1H), 3.50 (dd, *J* = 11.5, 8.0 Hz, 1H), 3.02 (dd, *J* = 11.5, 1.2 Hz, 1H), 2.77 (s, 3H), 2.45 – 2.28 (m, 1H), 1.06 (d, *J* = 6.8 Hz, 3H), 0.97 (d, *J* = 6.9 Hz, 3H); **^{13}C NMR** (101 MHz, $CDCl_3$) δ 203.4, 170.9, 71.40, 30.9, 30.5, 27.1, 19.2, 17.9; TLC (hexane/Et_2O 9:1, $KMnO_4$-stain) **R_f** = 0.25.

(*R*,4*E*,6*E*)-7-bromo-3-((*tert*-butyldimethylsilyl)oxy)-1-((*R*)-4-isopropyl-2-thioxothiazolidin-3-yl)hepta-4,6-dien-1-one (19)

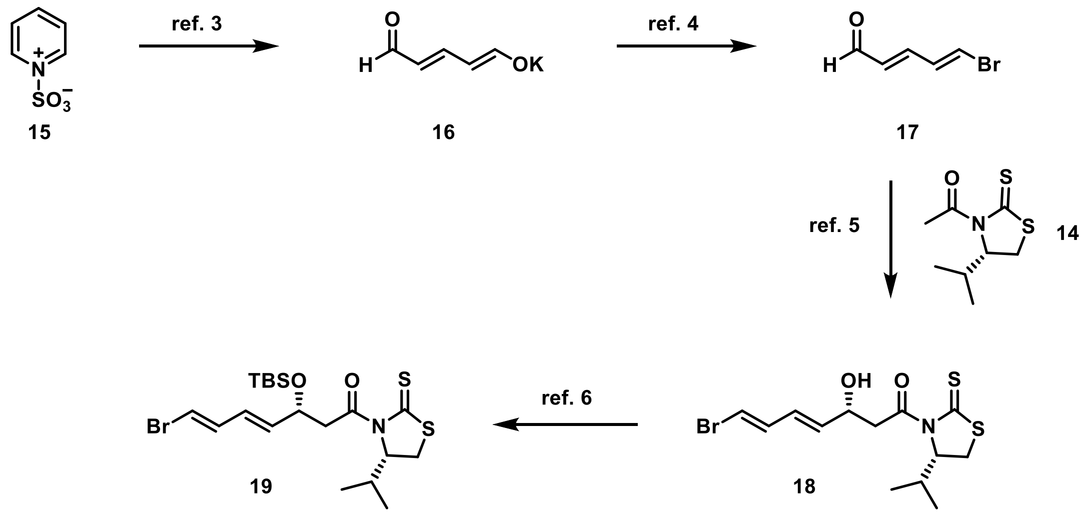

Thiazolidinethione **19** was prepared in four steps from commercially available pyridinium-1-sulfonate **15** as previously reported in the literature.[3-6] **Yield**: 28% over the four steps. All spectroscopic and physical data were in agreement with those reported in the literature.[6] **[α]** $^{20}_{D}$ = 265 (c = 0.40, $CHCl_3$); **^{1}H NMR** (400 MHz, $CDCl_3$) δ 6.69 (dd, *J* = 13.5, 10.9 Hz, 1H), 6.31 (d, *J* = 13.5 Hz, 1H), 6.15 (dd, *J* = 15.3, 10.9 Hz, 1H), 5.79 (dd, *J* = 15.4, 6.3 Hz, 1H), 5.08 – 4.98 (m, 1H), 4.79 – 4.69 (m, 1H), 3.64 (dd, *J* = 16.6, 7.9 Hz, 1H), 3.47 (dd, *J* = 11.5, 7.8 Hz, 1H), 3.21 (dd, *J* = 16.6, 4.5 Hz, 1H), 3.03 (dd, *J* = 11.4, 1.1 Hz, 1H), 2.43 – 2.29 (m, *J* = 6.9 Hz, 1H), 1.05 (d, *J* = 6.8 Hz, 3H), 0.97 (d, *J* = 7.0 Hz, 3H), 0.86 (s, 9H), 0.05 (s, 3H), 0.03 (s, 3H); **^{13}C NMR** (101 MHz, $CDCl_3$) δ 203.0, 171.0, 136.9, 127.5, 109.1, 71.8, 69.9, 46.3, 31.0, 30.9, 26.0 (3C), 19.3, 18.2, 18.0, -4.2, -4.8; TLC (hexane/EtOAc, 7:3, $KMnO_4$-stain) **R_f** = 0.56.

(*S*)-3-((*tert*-butyldimethylsilyl)oxy)pent-4-yn-1-ol (21)

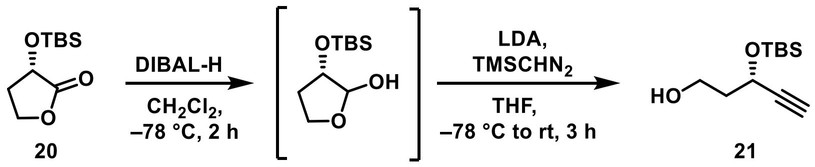

To a solution of TBS-lactone **20** (1.50 g, 6.90 mmol, 1.00 equiv.) in CH_2Cl_2 (75 mL) was added DIBAL-H (1.0 M in CH_2Cl_2, 8.30 mL, 8.30 mmol, 1.20 equiv.) at –78 °C. The reaction mixture was stirred for 2 h at this temperature and then quenched by addition of MeOH (10 mL). The solution was poured into a saturated aq. solution of Rochelle salt (potassium sodium tartrate) (100 mL) and vigorously stirred for 3 hours at room temperature. The layers were separated and the aq. layer was extracted (CH_2Cl_2, 3 x 50 mL). The combined organic layers were washed with brine (50 mL), dried ($MgSO_4$), filtered, and the solvent removed *in vacuo* to yield the crude lactol. Next, to a solution of LDA (1.0 M in hexane/THF, 16.6 mL, 16.6 mmol, 2.40 equiv.) in THF (18 mL) was added $TMSCHN_2$ (2.0 M in Et_2O, 4.14 mL, 8.28 mmol, 1.20 equiv.) at –78 °C and the reaction mixture was stirred for 30 min at the same temperature. The crude lactol in THF (20 mL) was carefully added and stirring was continued for 2 h. The reaction was warmed to rt, stirred for 30 min.[1] and then quenched by careful addition of a saturated aq. solution of NH_4Cl (15 mL). The layers were separated and the aqueous layer was extracted with Et_2O (3 × 50 mL). The combined organic layers were washed with brine (20 mL), dried ($MgSO_4$), filtered, and the solvent removed *in vacuo*. Alcohol **21** (832 mg, 3.88 mmol, 46%) was obtained after purification by column chromatography (heptane/EtOAc, 8:2) as a colorless oil. $[\alpha]_D^{20}$ = -55 (c = 0.11, $CHCl_3$); **^{1}H NMR** (400 MHz, $CDCl_3$) δ 4.61 (ddd, *J* = 7.0, 5.1, 2.1 Hz, 1H), 3.89 (ddd, *J* = 11.8, 7.6, 4.2 Hz, 1H), 3.75 (ddd, *J* = 10.9, 6.1, 4.5 Hz, 1H), 2.47 (bs, 1H), 2.42 (d, *J* = 2.1 Hz, 1H), 2.02 – 1.82 (m, 2H), 0.88 (s, 9H), 0.14 (s, 3H), 0.12 (s, 3H); **^{13}C NMR** (101 MHz, $CDCl_3$) δ 84.8, 73.2, 61.8, 59.8, 40.2, 25.8 (3C), 18.2, -4.5, -5.1; TLC (hexane/EtOAc 8:2, $KMnO_4$ stain) $\mathbf{R}_f$ = 0.21.

(*S*)-3-((*tert*-butyldimethylsilyl)oxy)pent-4-ynal (7)

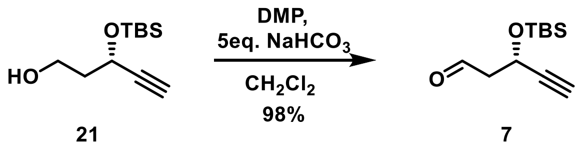

Alcohol **21** (529 mg, 2.47 mmol, 1.00 equiv.) dissolved in dry CH_2Cl_2 (22 mL) was added Dess-Martin periodinane (1.27 g, 2.99 mmol, 1.21 equiv.) and $NaHCO_3$ (s) (972 mg, 11.6 mmol, 4.69 equiv.). After

6 hours, the reaction mixture was quenched with a saturated solution of $Na_2S_2O_3$ (aq) (16 mL) and $NaHCO_3$ (aq) (16 mL). The layers were separated and the aqueous layer was extracted with Et_2O (3 × 10 mL). The combined organic extracts were washed with brine (10 mL) dried (Na_2SO_4) and concentrated *in vacuo.* The crude product was passed through a silica plug with hexane/EtOAc 8:2 as eluent to afford the title compound **7** as a colorless oil. **Yield**: 515 mg (98%). All spectroscopic and physical data were in agreement with those reported in the literature.[7] **^{1}H NMR** (400 MHz, $CDCl_3$) δ 9.84 – 9.81 (m, 1H), 4.86 (ddd, *J* = 7.0, 4.9, 2.2 Hz, 1H), 2.84 – 2.66 (m, 2H), 2.49 (d, *J* = 2.1 Hz, 1H), 0.88 (s, 9H), 0.17 (s, 3H), 0.13 (s, 3H); **^{13}C NMR** (101 MHz, $CDCl_3$) δ 200.2, 83.9, 73.9, 58.2, 51.5, 25.8, 18.2, -4.5, -5.1; TLC (EtOAc/hexane 1:4, $KMnO_4$ stain) **R_f** = 0.62.

Methyl (*R*,7*Z*,11*E*,13*E*)-14-bromo-10-((*tert*-butyldimethylsilyl)oxy)tetradeca-7,11,13-trienoate (9)

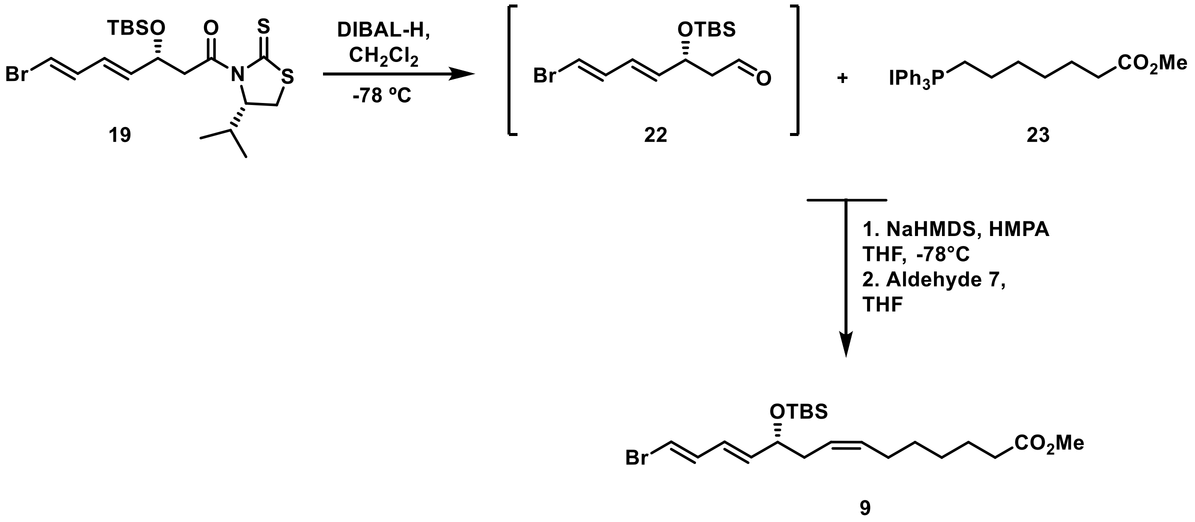

Following the procedure reported by Olivo and coworkers,[8] the protected thiazolidinethione **19** (578 mg, 1.21 mmol, 1.00 equiv.) was dissolved in CH_2Cl_2 (23 mL) followed by dropwise addition of DIBAL-H (1.0 M in CH_2Cl_2, 1.45 mmol, 1.20 equiv.) at -78 °C. After three h., additional DIBAL-H was added (1.0 M in CH_2Cl_2, 0.240 mmol, 0.198 equiv.). The mixture was allowed to stir for 30 min and then quenched with saturated $NaHCO_3$ (aq) (14 mL). The cooling bath was removed and solid Na-K tartrate (~ 0.400 g) (Rochelle salt) was added and stirring was continued for another 45 min. Et_2O (35 mL) was added. The layers were separated and the aq. layer was extracted with Et_2O (3 × 30 mL). The combined organic layers were dried (Na_2SO_4) and concentrated *in vacuo*. The residue was purified by column chromatography on silica gel (hexane/EtOAc 95:5) **R_f** = 0.24, and concentrated *in vacuo*, but not to dryness. Commercially available Wittig salt **23** (670 mg, 1.26 mmol, 1.00 equiv.) in THF (16 mL) and HMPA (1.7 mL) was slowly added NaHMDS (0.6 M in THF, 2.1 mL, 1.04 equiv.) at -78 °C and then

stirred for 15 min at 0 °C. The purified aldehyde **22** was added. The solution was allowed to slowly warm up to room temperature in the dry ice/acetone bath for 24 h before it was quenched with phosphate buffer (12 mL, pH = 7.2). Et_2O (15 mL) was added and the phases were separated. The aqueous phase was extracted with Et_2O (2 × 15 mL) and the combined organic layers were dried (Na_2SO_4), before concentrated *in vacuo.* The crude product was purified by column chromatography on silica (hexane/EtOAc 95:5) to afford the title compound **9** as a clear oil. **Yield**: 386 mg (45-72% over two steps). All spectroscopic and physical data were in agreement with those reported in the literature.[9] $[\alpha]_D^{20}$ = -18.0 (c = 0.090, MeOH); **^{1}H NMR** (400 MHz, $CDCl_3$) δ 6.68 (dd, *J* = 13.4, 10.9 Hz, 1H), 6.27 (d, *J* = 13.5 Hz, 1H), 6.09 (dd, *J* = 15.2, 10.9 Hz, 1H), 5.71(dd, *J* = 15.3, 9.6 Hz, 1H), 5.48−5.29 (m, 2H), 4.17 – 4.11 (m, 1H), 3.67 (s, 3H), 2.34 – 2.16 (m, 4H), 2.00 (q, *J* = 6.8 Hz, 2H), 1.62 (p, *J* = 7.5 Hz, 2H), 1.41 – 1.26 (m, 4H), 0.89 (s, 9H), 0.04 (s, 3H), 0.02 (s, 3H); **^{13}C NMR** (101 MHz, $CDCl_3$) δ 174.4, 138.1, 137.2, 131.9, 126.6, 125.2, 108.2, 72.7, 51.6, 36.3, 34.2, 29.4, 29.0, 27.4, 26.0 (3C), 25.0, 18.4, -4.4, -4.6; TLC (hexane/EtOAc 95:5, $KMnO_4$ stain) $\mathbf{R}_f$ = 0.33.

^{1}H NMR and ^{13}C NMR spectra of compounds

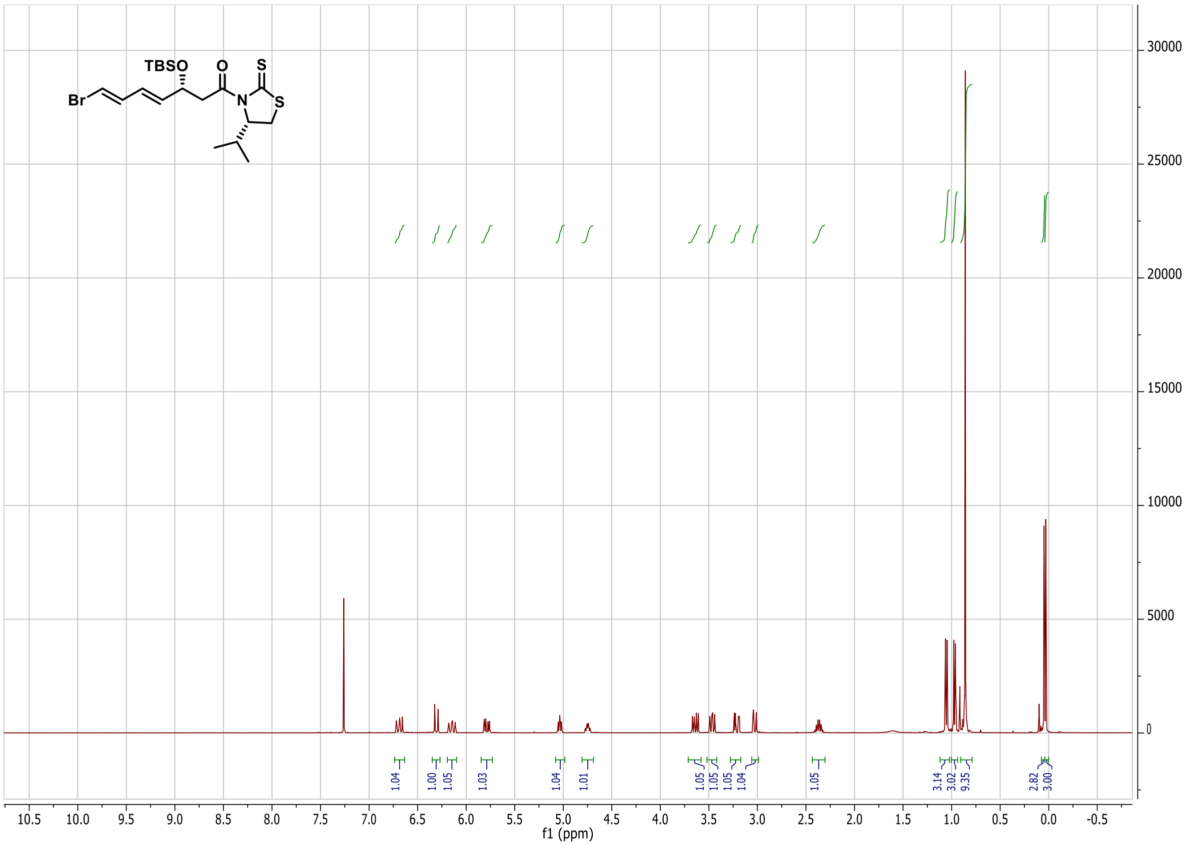

Figure S-1. ^{1}H NMR spectrum of compound **19**.

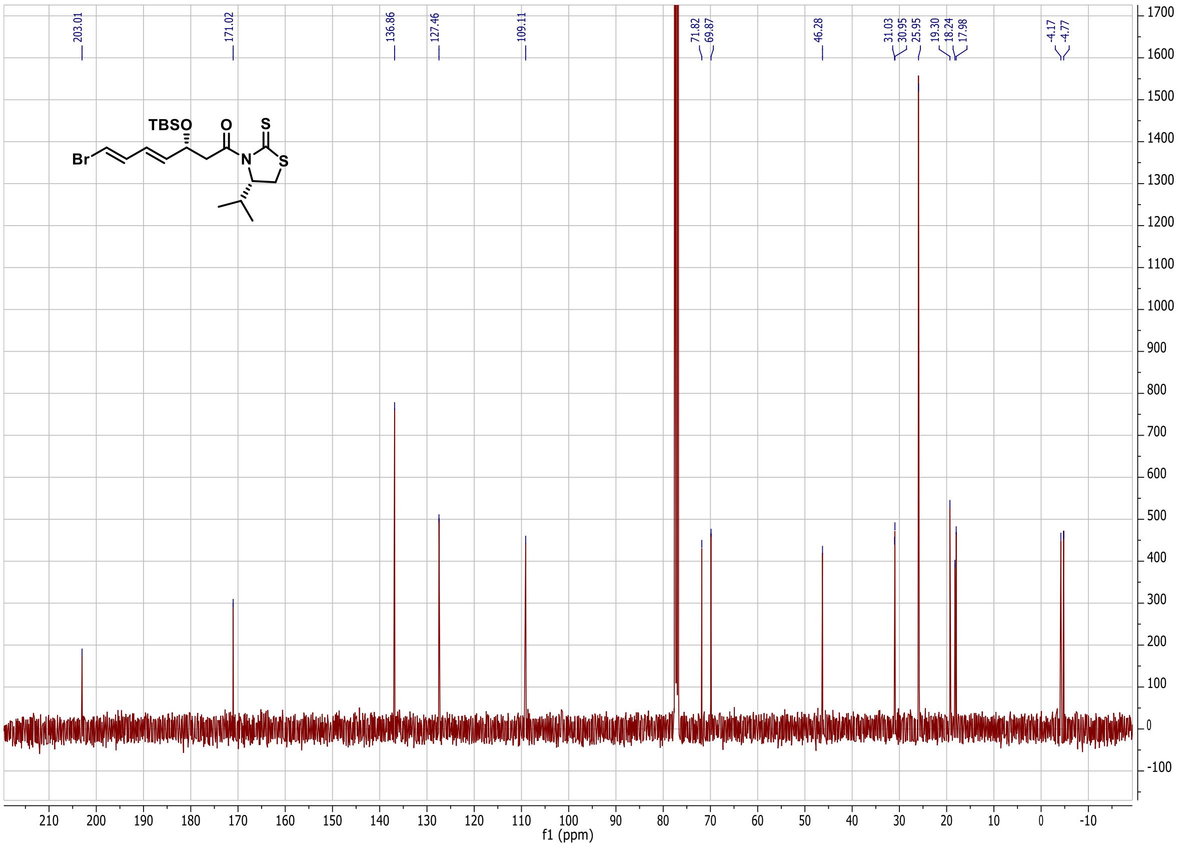

Figure S-2. ^{13}C NMR spectrum of compound **19**.

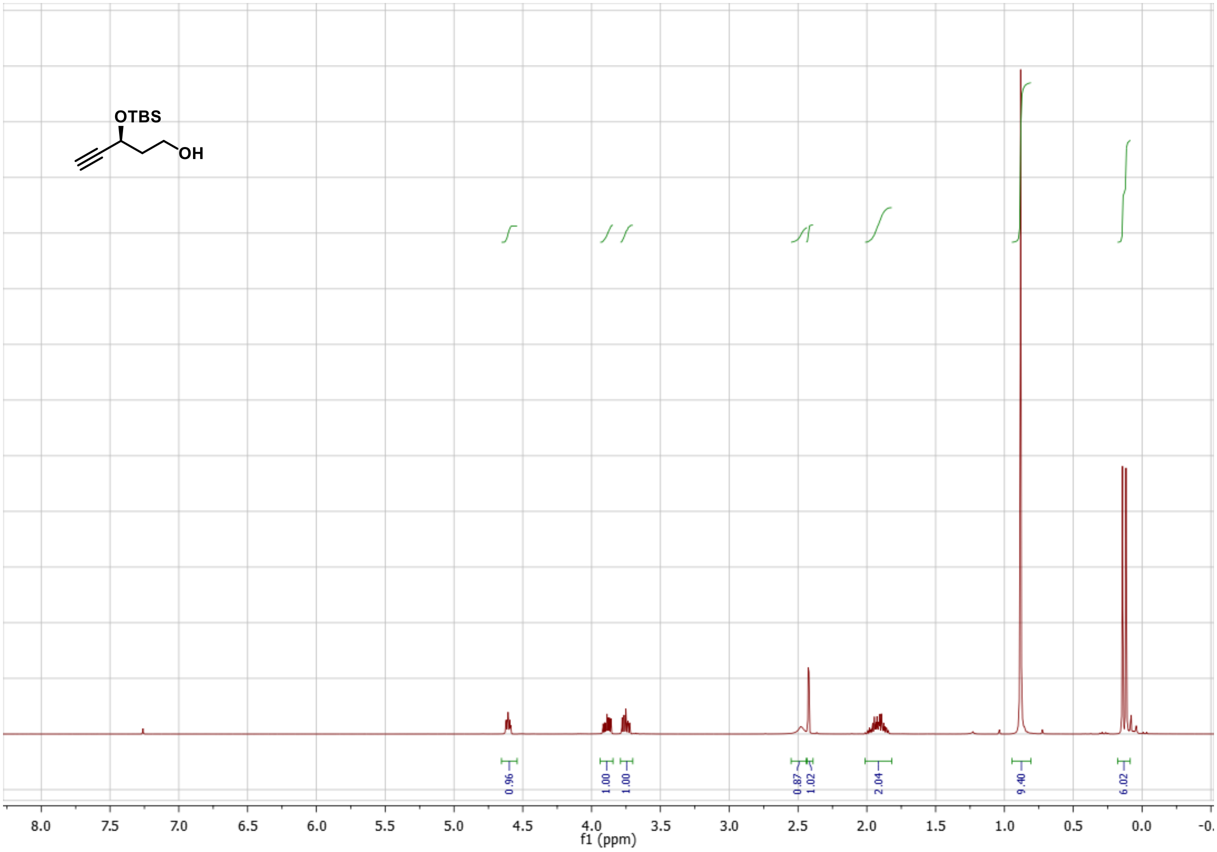

Figure S-3. ^{1}H NMR spectrum of compound **21**.

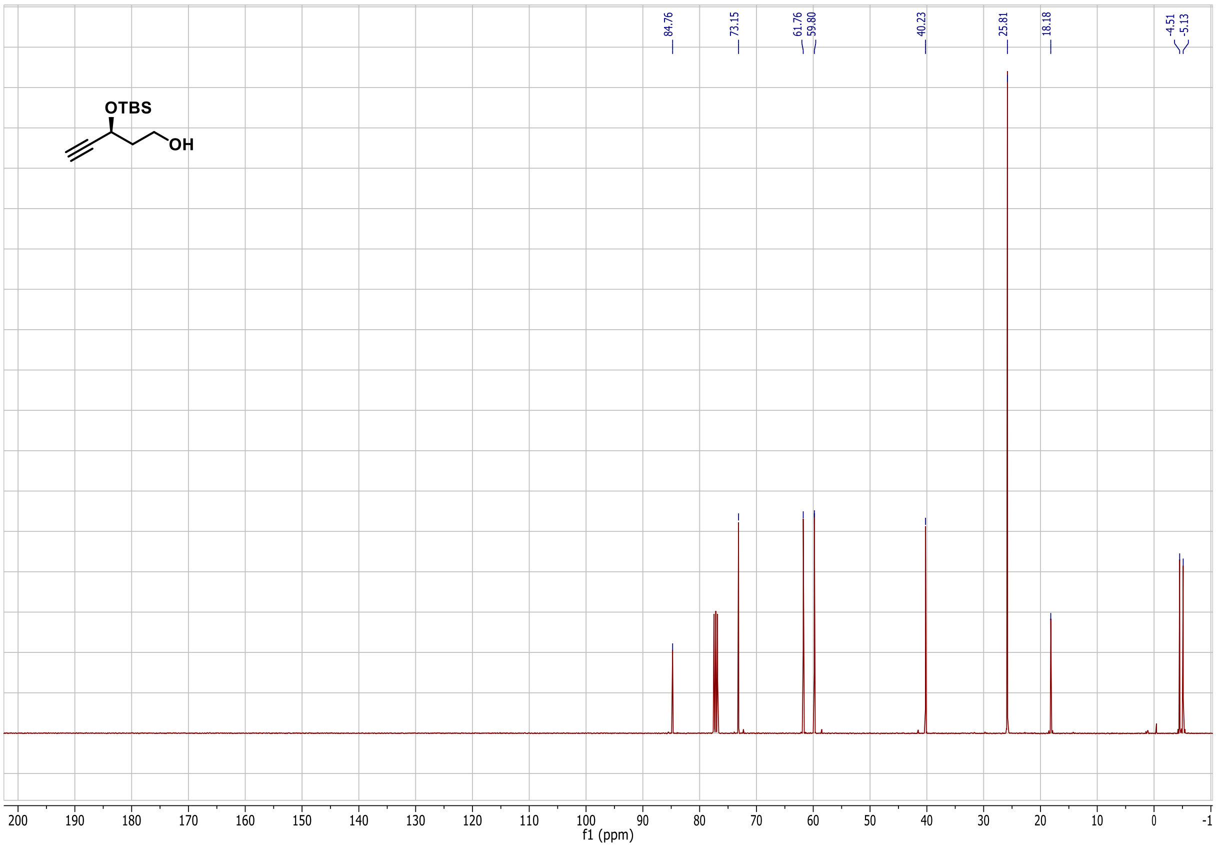

Figure S-4. ^{13}C NMR spectrum of compound **21**.

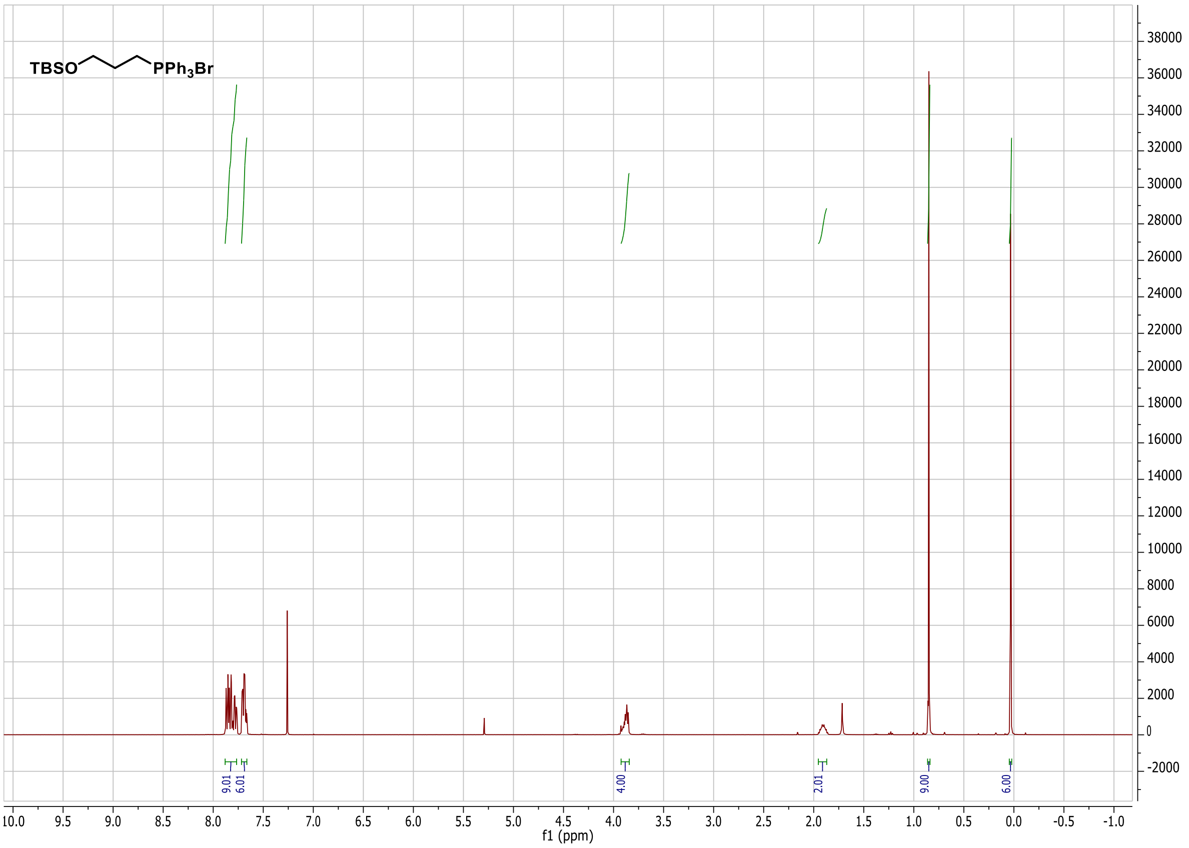

Figure S-5. ^{1}H NMR spectrum of compound **6**.

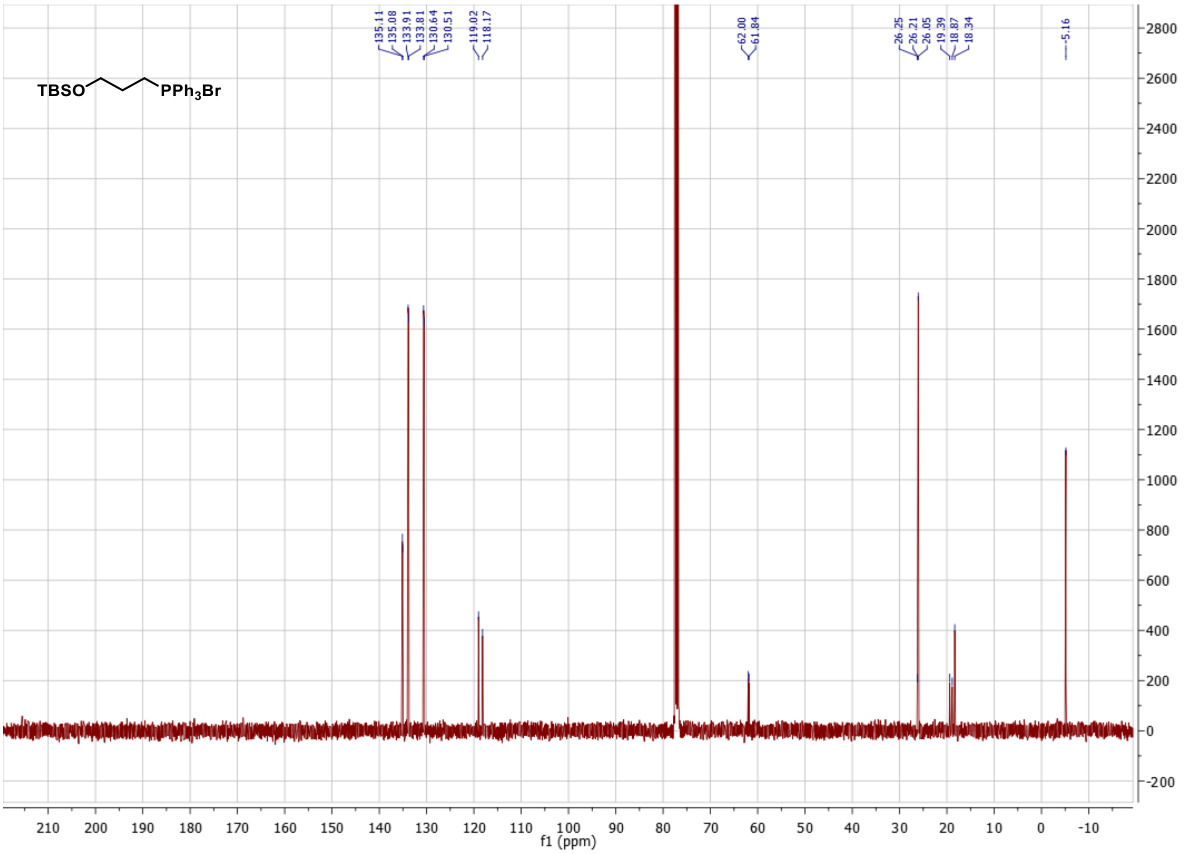

Figure S-6. ^{13}C NMR spectrum of compound **6**.

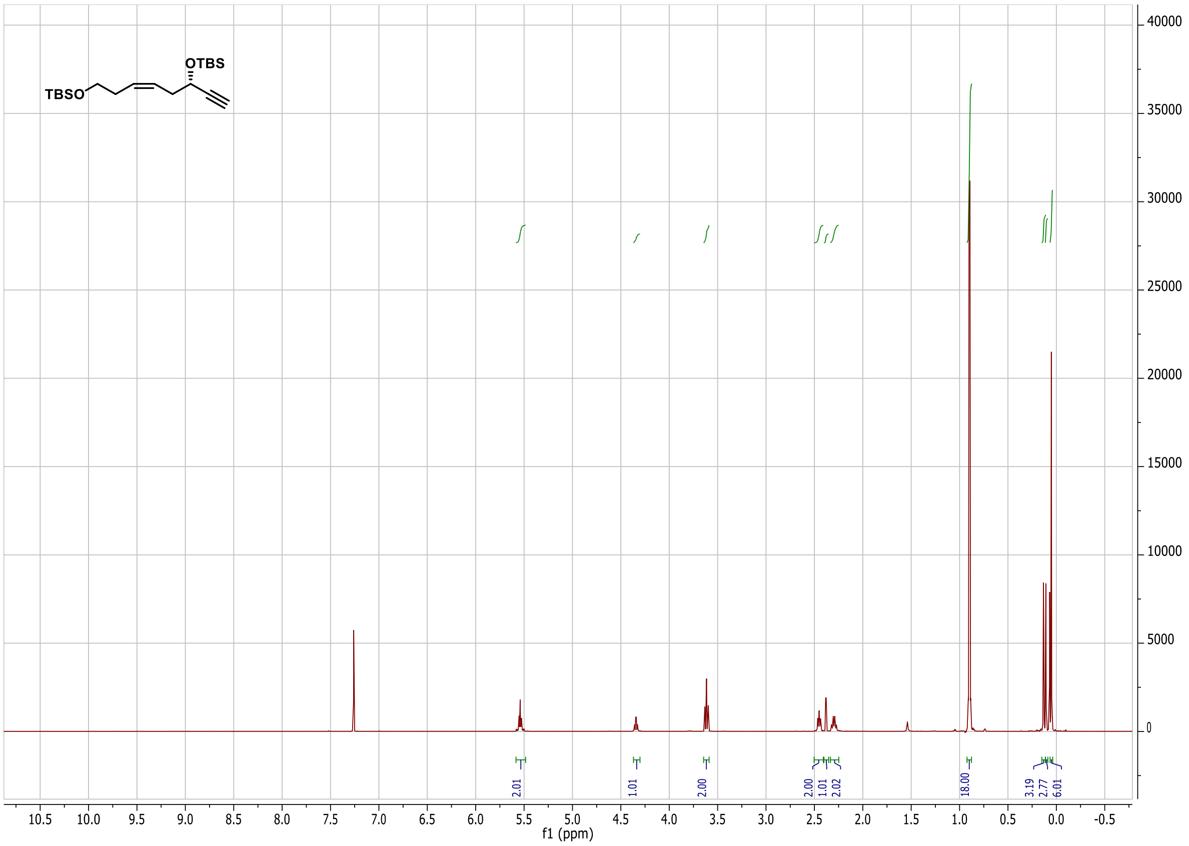

Figure S-7. ^{1}H NMR spectrum of compound **8**.

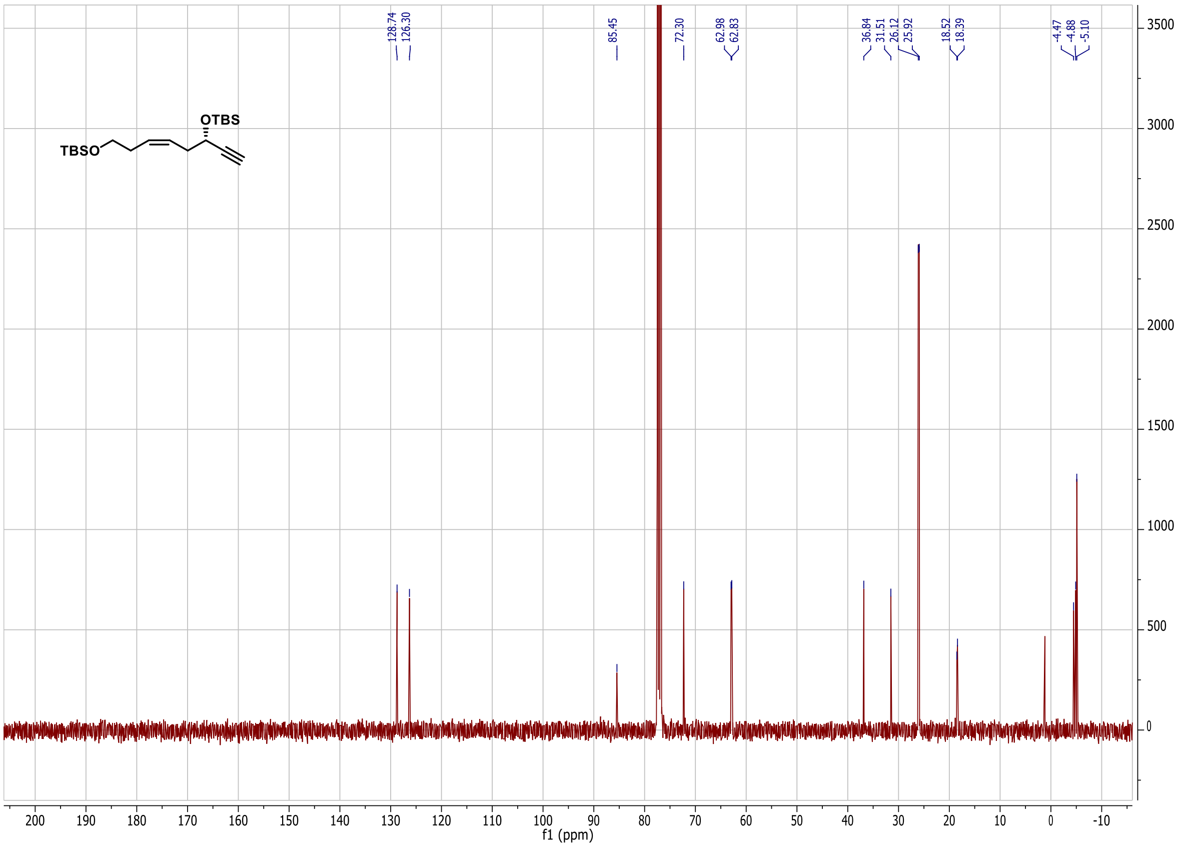

Figure S-8. ^{13}C NMR spectrum of compound **8**.

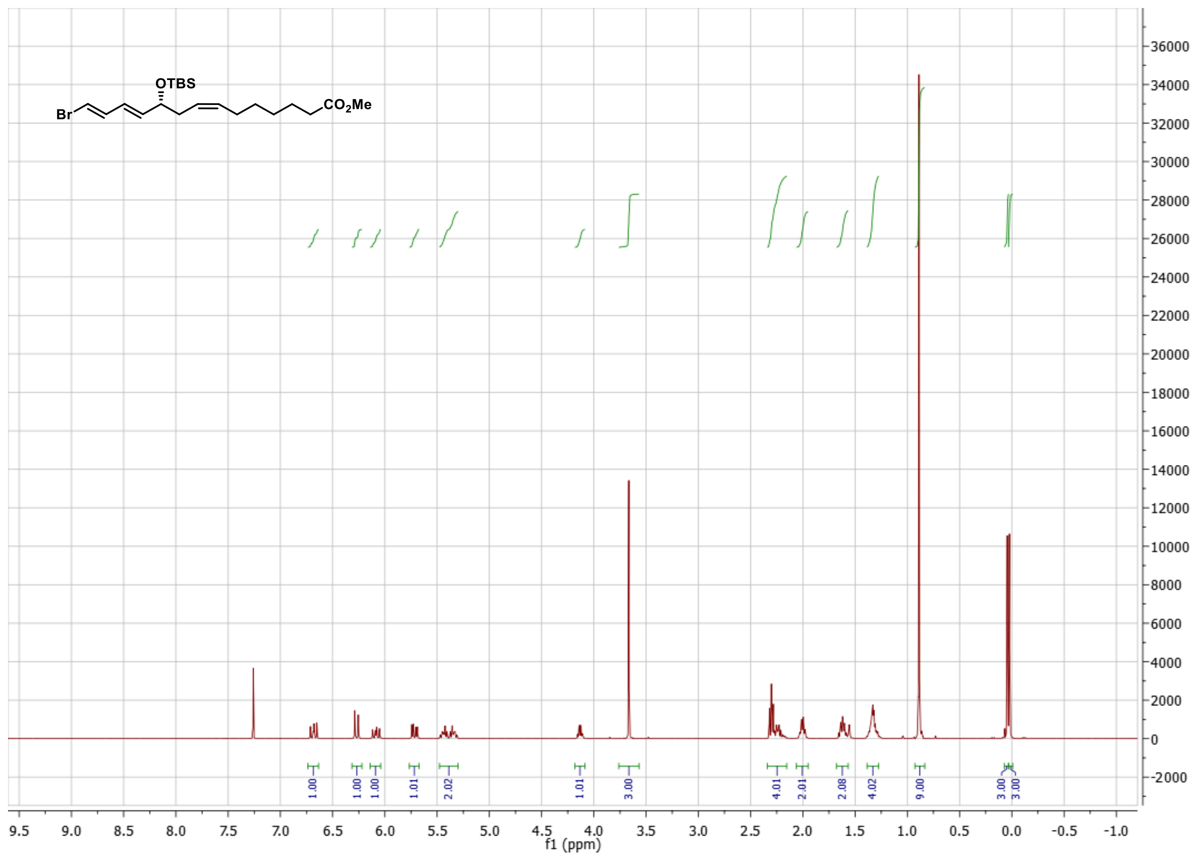

Figure S-9. ^{1}H NMR spectrum of compound **9**.

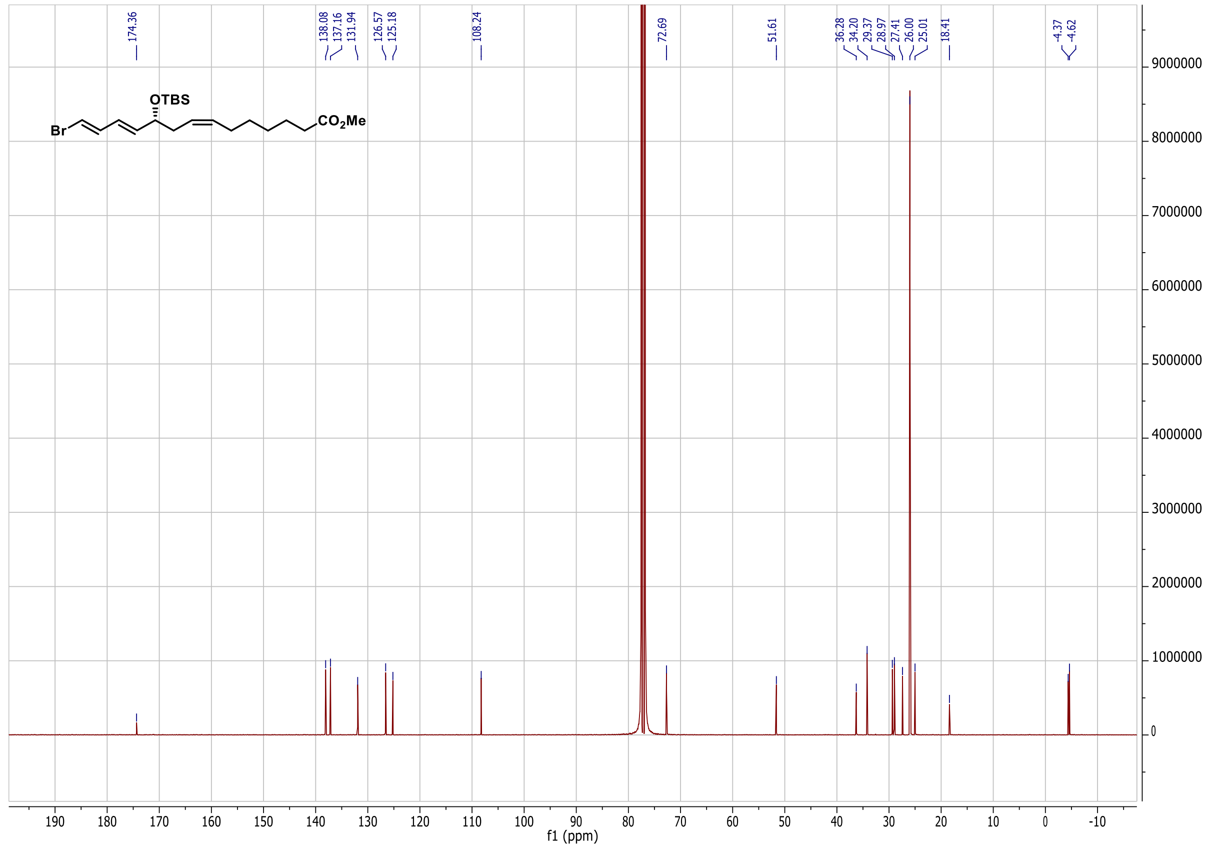

Figure S-10. ^{13}C NMR spectrum of compound **9**.

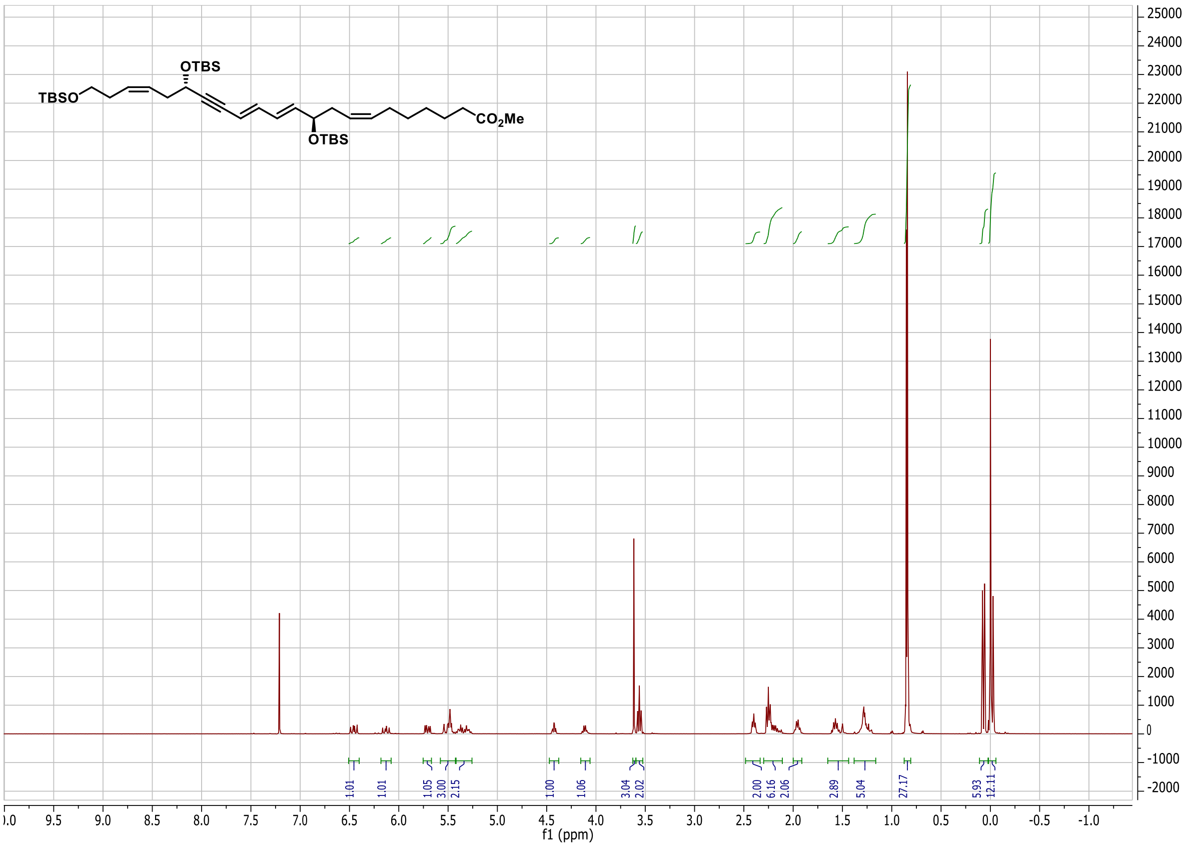

Figure S-11. ^{1}H NMR spectrum of compound **10**.

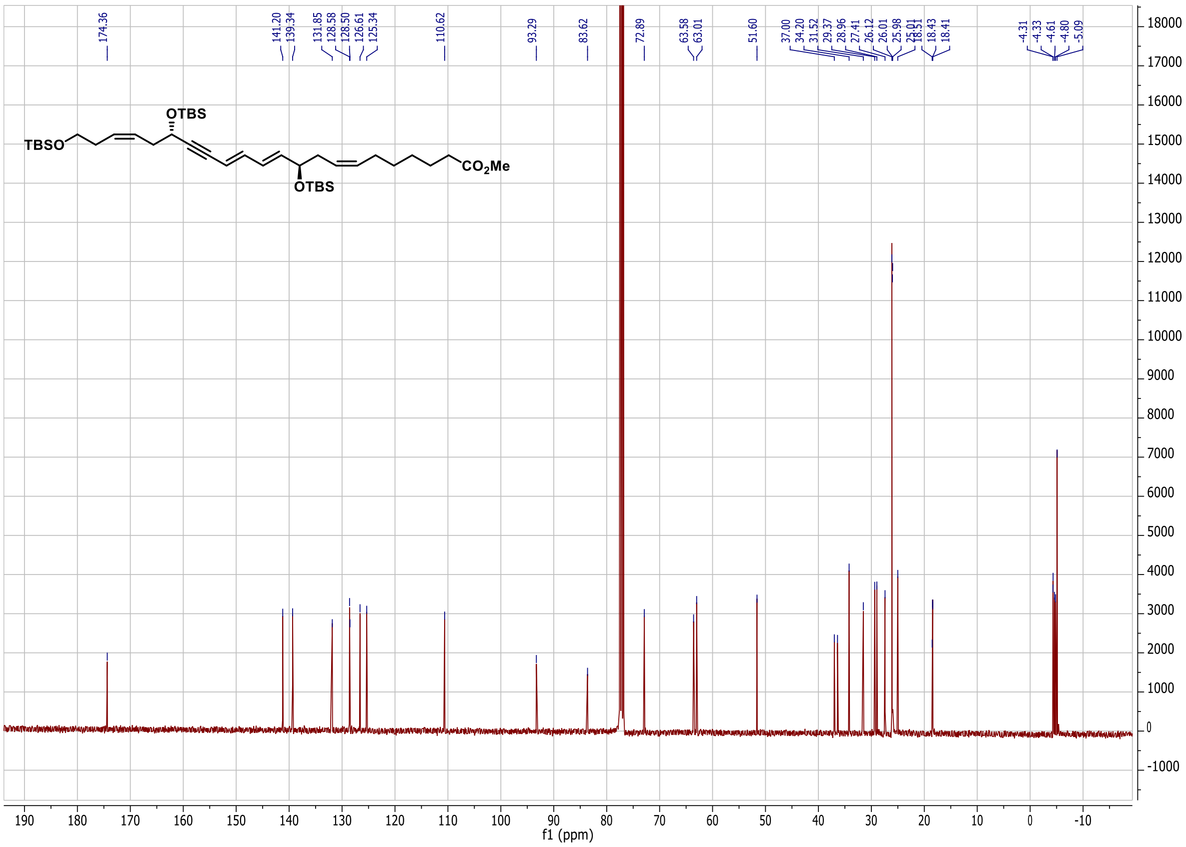

Figure S-12. ^{13}C NMR spectrum of compound **10**.

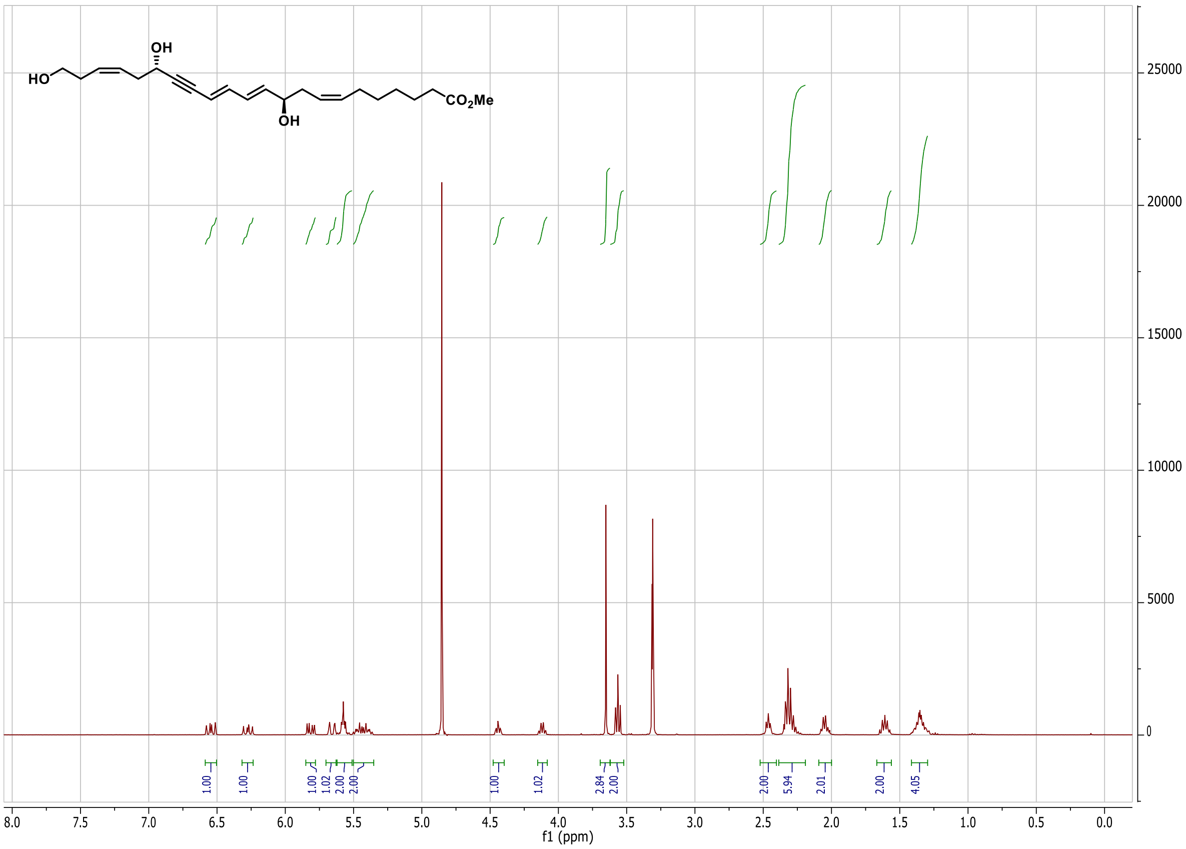

Figure S-13. ^{1}H NMR spectrum of compound **11**.

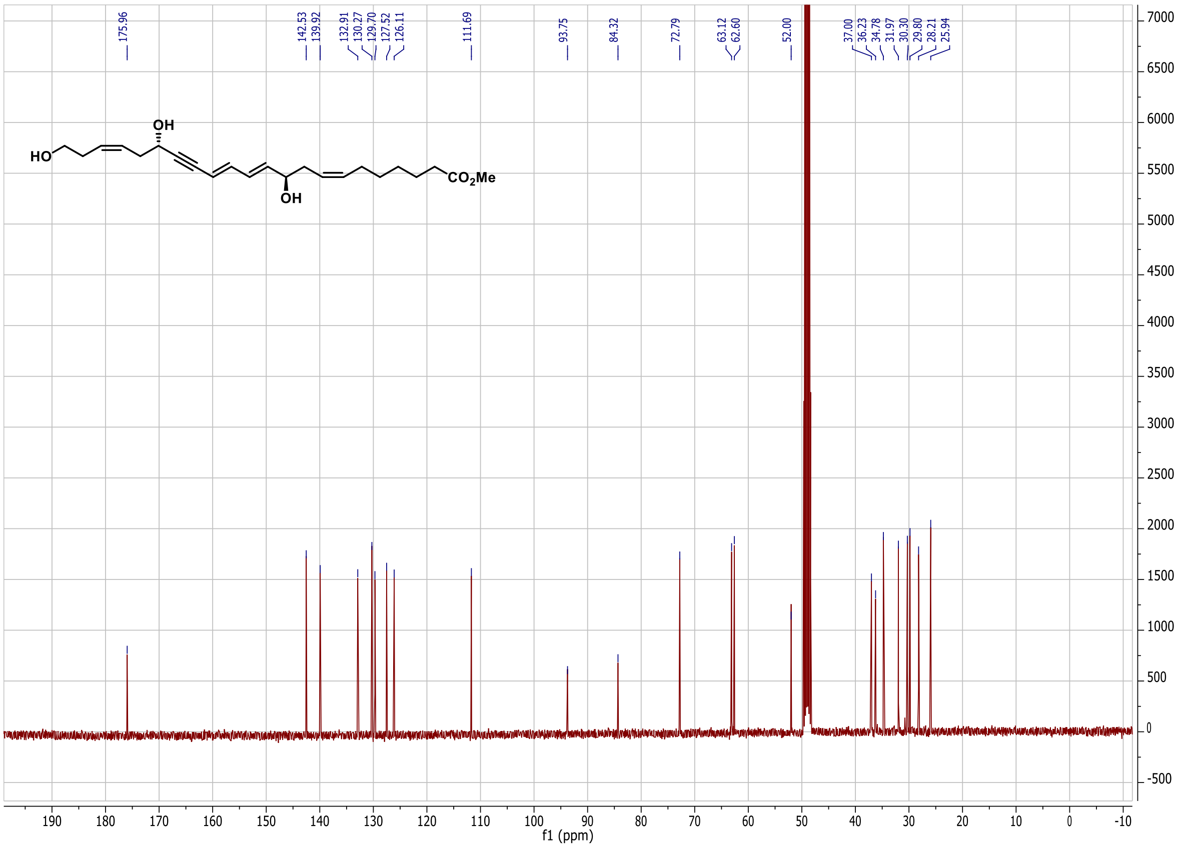

Figure S-14. ^{1}H NMR spectrum of compound **11**.

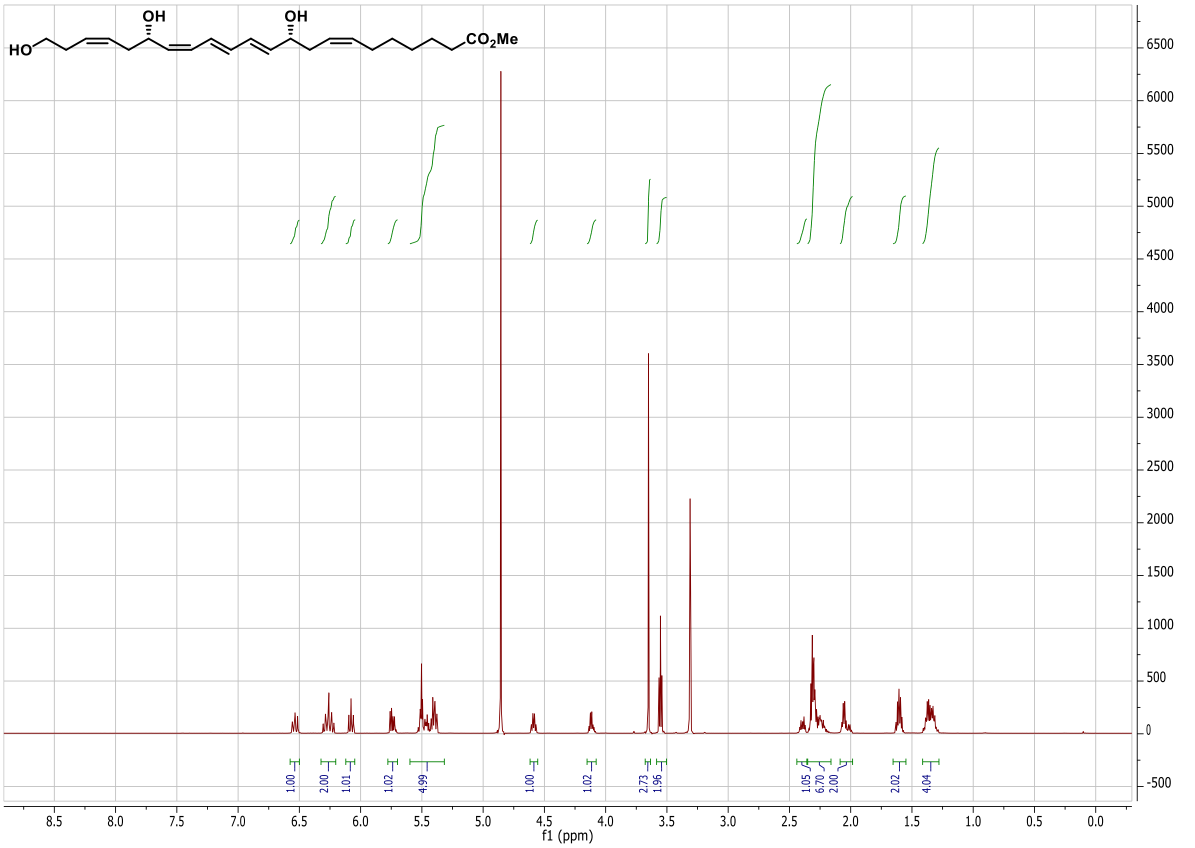

Figure S-15. ^{1}H NMR spectrum of 22-OH-PD1$_{n\text{-}3\ DPA}$ methyl ester (**12**).

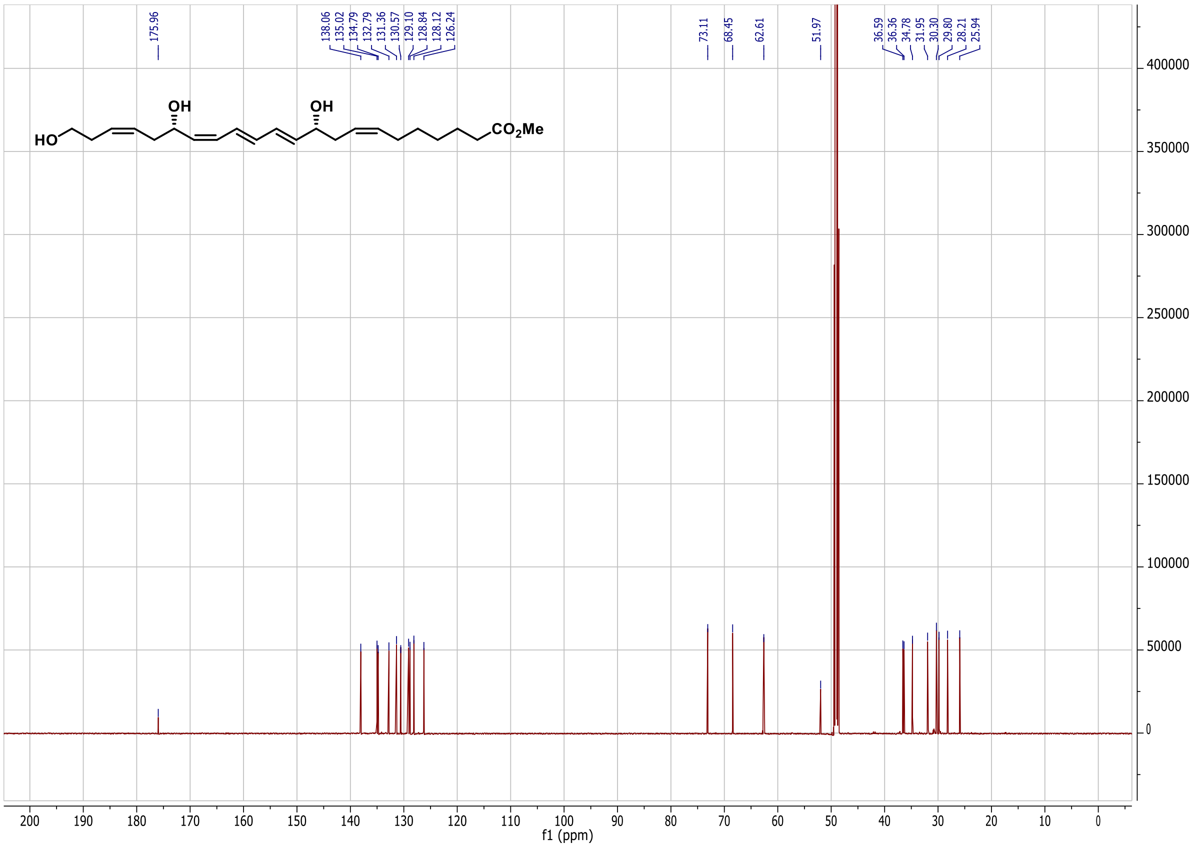

Figure S-16. ^{13}C NMR spectrum of 22-OH-PD1$_{n\text{-}3\ DPA}$ methyl ester (**12**).

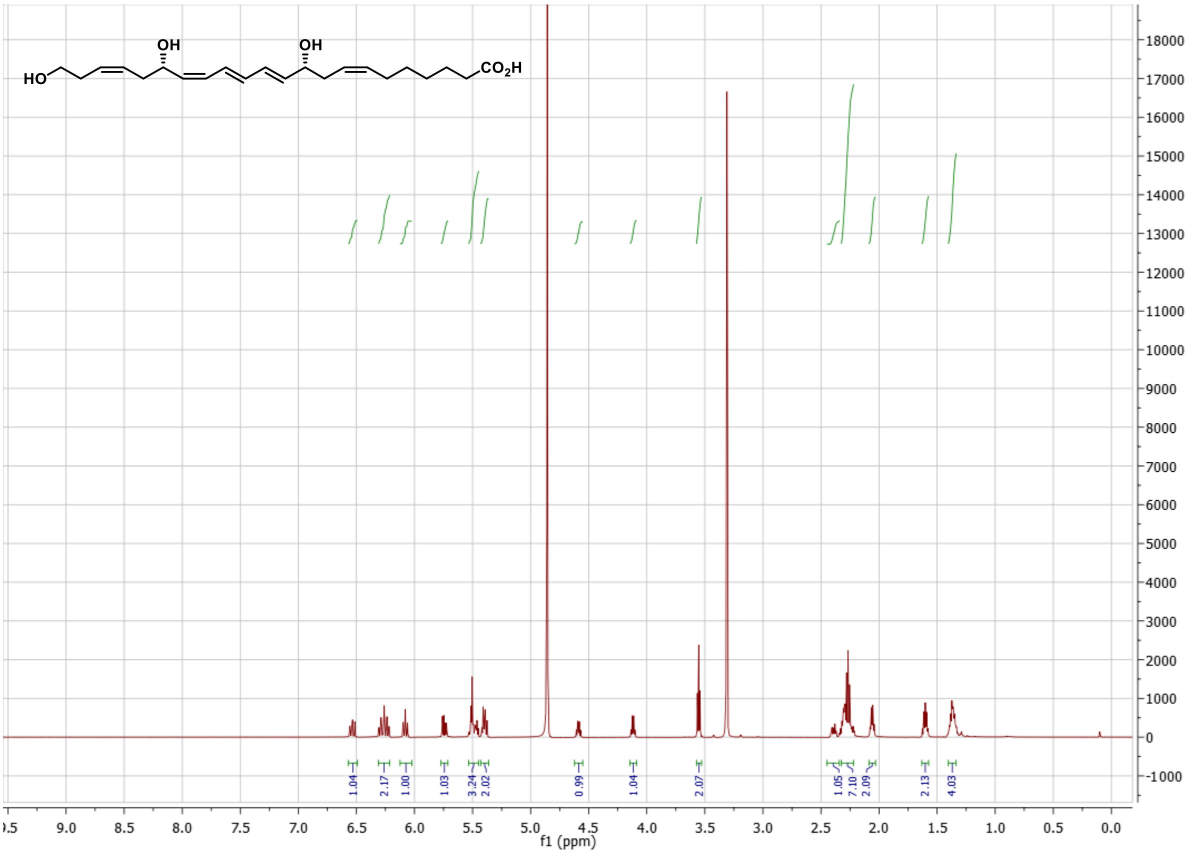

Figure S-17. ^{1}H NMR spectrum of 22-OH-PD1$_{n\text{-}3\ DPA}$ (**5**).

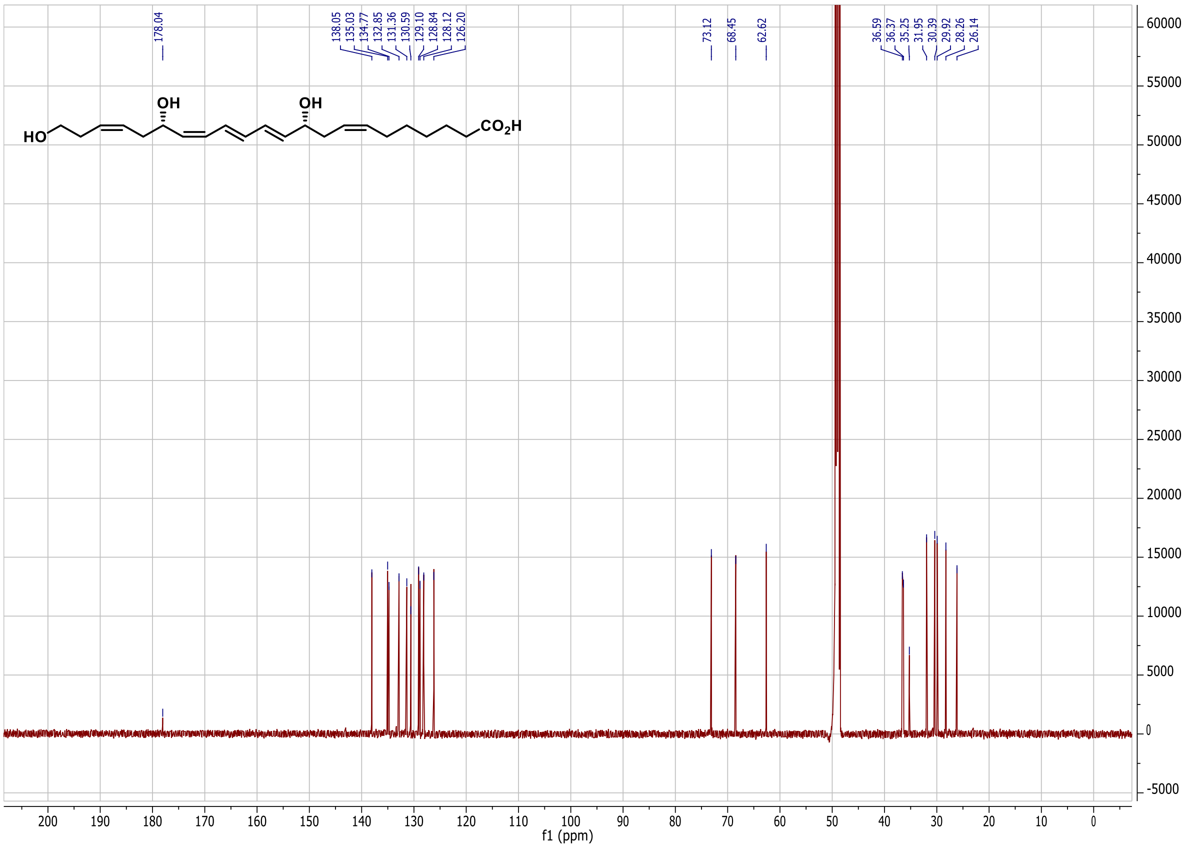

Figure S-18. ^{13}C NMR spectrum of 22-OH-PD1$_{n\text{-}3\ DPA}$ (**5**).

HPLC chromatograms

Data File C:\CHEM32\1\DATA\SILJE\JIN36-F6.D
Sample Name: JIN36-F6

Acq. Operator : Silje
Acq. Instrument : Instrument 1 Location : Vial 1
Injection Date : 24.05.2018 13:13:24
Inj Volume : 15 µl
Method : C:\CHEM32\1\METHODS\DGTHMTST.M
Last changed : 24.05.2018 10:40:55 by Silje
(modified after loading)
Method Info : Column thermostat functional test method

Sample Info : Flash 5 metylester 22-OH-PD1n-3. 60:40, MeOH:H2O. 1ml/m in, 271 nm, C-18 kolonne

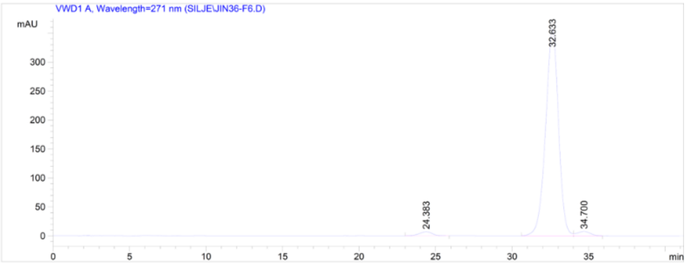

Area Percent Report

Sorted By : Signal
Multiplier : 1.0000
Dilution : 1.0000
Sample Amount : 1.00000 [ng/ul] (not used in calc.)
Use Multiplier & Dilution Factor with ISTDs

Signal 1: VWD1 A, Wavelength=271 nm

Peak #	RetTime [min]	Type	Width [min]	Area mAU *s	Height [mAU]	Area %
1	24.383	BB	0.6846	390.51266	6.71915	1.7983
2	32.633	BV	0.8864	2.08952e4	356.39899	96.2198
3	34.700	VB	0.6923	430.39163	7.44101	1.9819
Totals :				2.17161e4	370.55914	

Figure S-19. HPLC chromatogram of 22-OH-PD1$_{\text{n-3 DPA}}$ methyl ester (**12**).

Data File C:\CHEM32\1\DATA\SILJE\JIN40TM-ACID.D
Sample Name: JIN40TM-ACID

Acq. Operator : silje
Acq. Instrument : Instrument 1 Location : Vial 1
Injection Date : 20.06.2018 16:04:13
Inj Volume : 10 µl
Acq. Method : C:\CHEM32\1\METHODS\DGTHMTST.M
Last changed : 20.06.2018 15:55:44 by silje
(modified after loading)
Analysis Method : C:\CHEM32\1\METHODS\DGTHMTST.M
Last changed : 21.06.2018 13:42:34 by Jorn
(modified after loading)
Method Info : Column thermostat functional test method

Sample Info : JIN40TM-ACIDsyre, målmolekyl 271 nm, 1 mL/min, 65:20:20 metanol vann, 10mM maursyre, C-18, 10mikroliter injeks jon

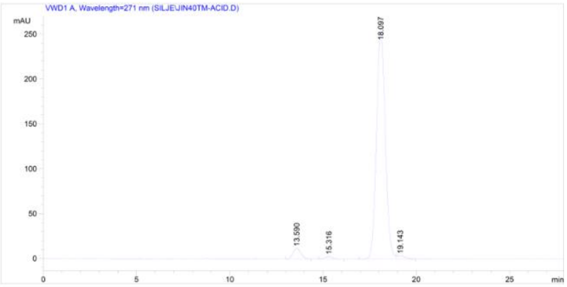

Area Percent Report

Sorted By : Signal
Multiplier : 1.0000
Dilution : 1.0000
Sample Amount : 1.00000 [ng/ul] (not used in calc.)
Use Multiplier & Dilution Factor with ISTDs

Signal 1: VWD1 A, Wavelength=271 nm

Peak #	RetTime [min]	Type	Width [min]	Area mAU *s	Height [mAU]	Area %
1	13.590	BB	0.4379	350.90503	11.84972	3.9367
2	15.316	BB	0.3232	77.37843	2.83119	0.8681
3	18.097	BV	0.5033	8372.52051	257.37936	93.9286
4	19.143	VB	0.3589	112.90607	3.70745	1.2667

Figure S-20. HPLC chromatogram of 22-OH-PD1$_{\text{n-3 DPA}}$ (**5**).

Lipid Mediator Metabololipidomics

Matching of synthetic **5** with endogenous products was conducted as previously reported.[10] Summarily, biological samples were subject to C18 solid-phase extraction. Prior to sample extraction, d_4-LTB_4, (500 pg), d_5-RvE1 (100 pg), were added as internal standards. Extracted samples were analyzed using QTrap 6500+ (ABSciex) MS system, coupled with a Shimadzu SIL-20AC HT autosampler and LC-20AD LC pumps. Agilent C18 Poroshell column (150 mm × 4.6 mm × 2.7 µm) was used to profile lipid mediators. The gradient was initiated at 20:80:0.01 (vol/vol/vol) methanol/water/acetic acid for 0.2 min this was ramped to 50:50:0.01 (vol/vol/vol) over 12 s, maintained for 2 min, then ramped to 80:20:0.01 (vol/vol/vol) over 9 min, and maintained for 3.5 min. The ratio was then ramped to 98:2:0.01 (vol/vol/vol) for 5.5 min. The flow rate was kept at 0.5 mL/min throughout elution.

Mediator identity was established using multiple reaction monitoring (MRM) using signature parent ion (Q1) and characteristic daughter ion (Q3) pairs to match retention time of the biological material to synthetic material (**5**). Then, using an Enhanced Product Ion (EPI) scan a minimum of six diagnostic ions were used to confirm identity, in accordance with published criteria.[10]

Matching of synthetic 22-OH-PD1$_{n-3\ DPA}$ with material formed in human monocytes incubated with PD1$_{n-3\ DPA}$

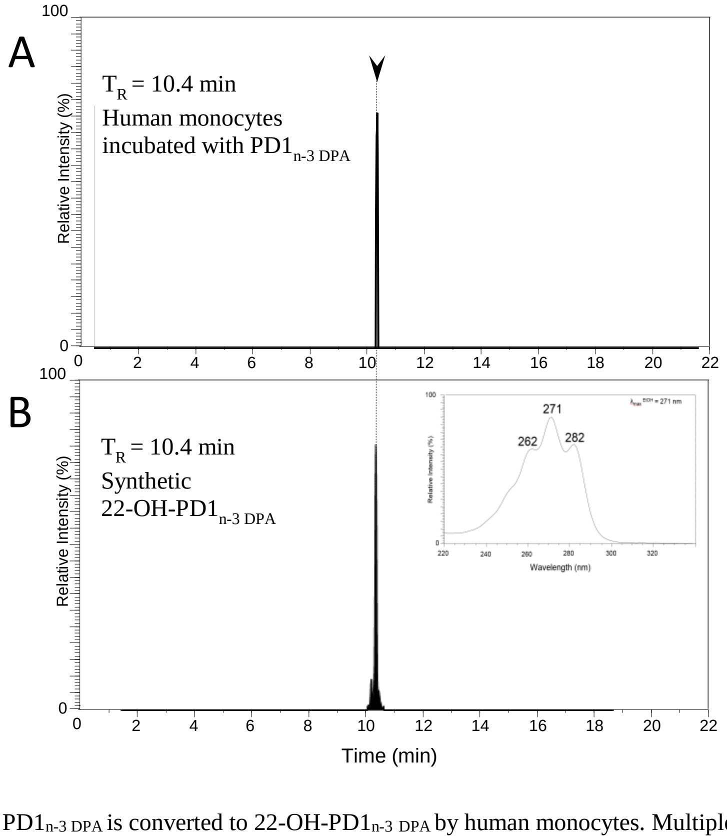

Figure S-21. PD1$_{n-3\ DPA}$ is converted to 22-OH-PD1$_{n-3\ DPA}$ by human monocytes. Multiple reaction monitoring chromatograms for *m/z* 377>361 of the products obtained from (A) Human monocytes incubated with PD1$_{n-3\ DPA}$. (B) Synthetic 22-OH-PD1$_{n-3\ DPA}$.

MS-MS fragmentation spectrums of 22-OH-PD1$_{n\text{-}3\ DPA}$

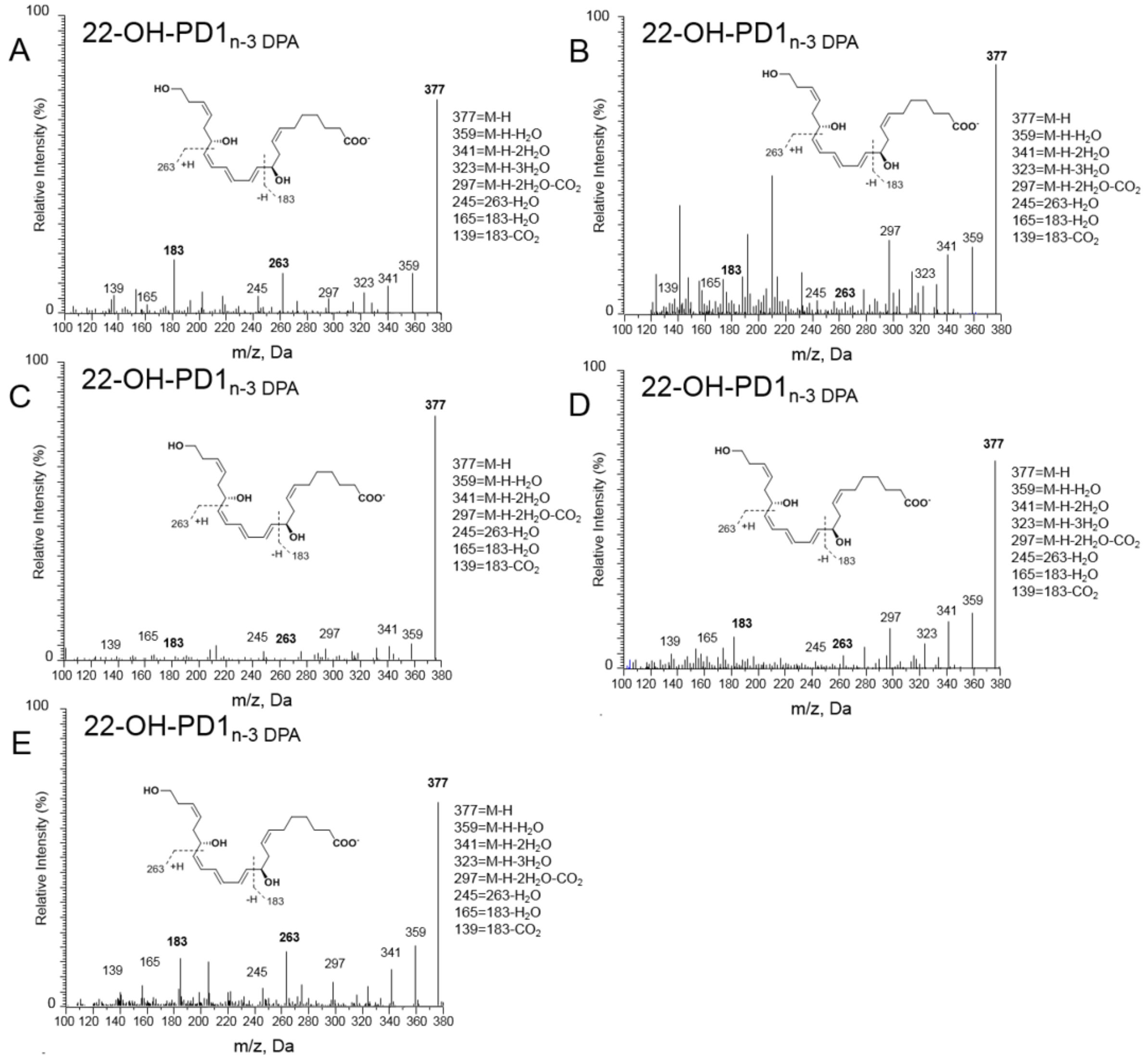

Figure S-22. MS-MS spectrum employed for identification of **5** obtained from (A) Synthetic material, (B) Human serum, (C) Human neutrophils, (D) Human monocytes, (E) Human neutrophils incubated with **2**.

UV-VIS spectra

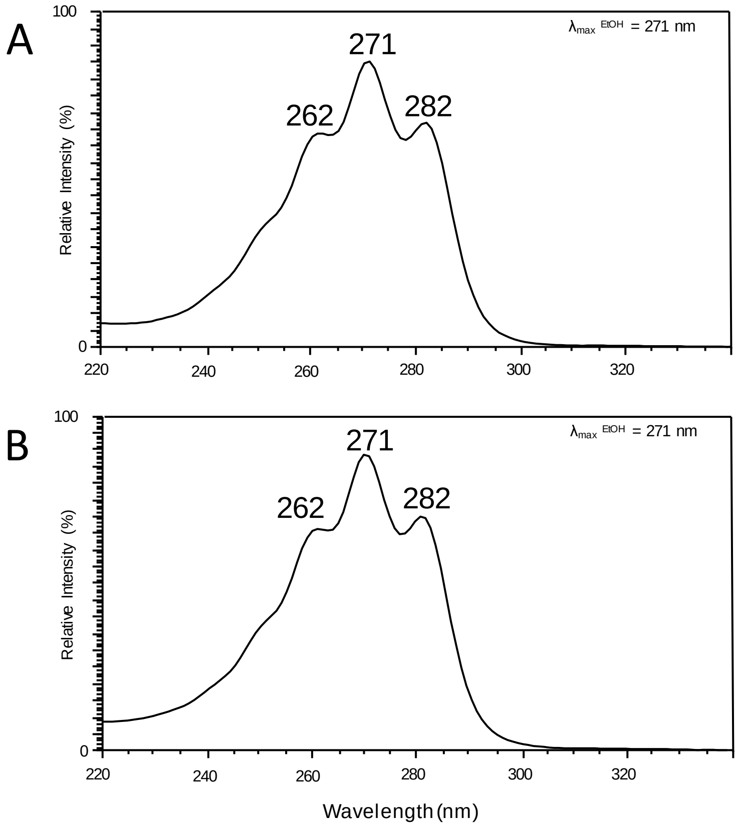

Figure S-23. UV-VIS absorption spectra of (A) synthetic 22-OH-PD1$_{n\text{-}3\ DPA}$ and (B) PD1$_{n\text{-}3\ DPA}$ in ethanol.

References

1. Delaunay, D.; Toupet, L.; Corre, M. L., *J. Org. Chem.* **1995**, *60* (20), 6604.
2. Nagao, Y.; Dai, W. M.; Ochiai, M.; Tsukagoshi, S.; Fujita, E., *J. Org. Chem.* **1990**, *55* (4), 1148.
3. Becher, J., *Org Synth.* **1979**, *59,* 79.
4. Soullez, D.; Plé, G.; Duhamel, L., *J. Chem. Soc., Perkin Transactions 1.* **1997**, (11), 1639.
5. Romero-Ortega, M.; Colby, D. A.; Olivo, H. F., *Tetrahedron Lett.* **2002**, *43,* 6439.
6. Corey, E. J.; Cho, H.; Rucker, C.; Hua, D. H., *Tetrahedron Lett.* **1981**, *22,* 3455.
7. Tungen, J. E.; Aursnes, M.; Dalli, J.; Arnardottir, H.; Serhan, C. N.; Hansen, T. V., *Chem. - Eur. J.* **2014**, *20,* 14575.
8. Tello-Aberto, R.; Ochoa-Teran, A.; Olivo, H. F., *Tetrahedron Lett.* **2006**, *47,* 5915.
9. Aursnes, M.; Tungen, J. E.; Vik, A.; Colas, R.; Cheng, C. Y.; Dalli, J.; Serhan, C.N.; Hansen, T.V., *J. Nat. Prod.* **2014**, *77* (4), 910.
10. Dalli, J.; Winkler, J. W.; Colas, R. A.; Arnadottir, H.; Cheng, C. Y.; Chiang, N.; Petasis, N. A.; Serhan, C. N., *Chem. Biol.* **2013**, *20* (2), 188.
